# In-depth characterization of a new patient-derived xenograft model for metaplastic breast carcinoma to identify viable biologic targets and patterns of matrix evolution within rare tumor types

**DOI:** 10.1007/s12094-021-02677-8

**Published:** 2021-08-09

**Authors:** M. D. Matossian, T. Chang, M. K. Wright, H. E. Burks, S. Elliott, R. A. Sabol, H. Wathieu, G. O. Windsor, M. S. Alzoubi, C. T. King, J. B. Bursavich, A. M. Ham, J. J. Savoie, K. Nguyen, M. Baddoo, E. Flemington, O. Sirenko, E. F. Cromwell, K. L. Hebert, F. Lau, R. Izadpanah, H. Brown, S. Sinha, J. Zabaleta, A. I. Riker, K. Moroz, L. Miele, A. H. Zea, A. Ochoa, B. A. Bunnell, B. M. Collins-Burow, E. C. Martin, M. E. Burow

**Affiliations:** 1 Department of Medicine, Section of Hematology & Medical Oncology, Tulane University School of Medicine, New Orleans, LA USA; 2 Tulane Center for Stem Cell Research and Regenerative Medicine, New Orleans, LA USA; 3 Department of Biological and Agricultural Engineering, Louisiana State University, Baton Rouge, LA USA; 4 Cancer Genetics Program, Tulane University School of Medicine, New Orleans, LA USA; 5 Molecular Devices LLC, San Jose, CA USA; 6 Protein Fluidics Inc, Burlingame, CA USA; 7 Department of Surgery, Section of Plastic & Reconstructive Surgery, Louisiana State University Health Sciences Center, New Orleans, LA USA; 8 Tulane Cancer Center, New Orleans, LA USA; 9 Innogenomics Technologies LLC, New Orleans, LA USA; 10 Department of Pediatrics, Louisiana State University Health Sciences Center, New Orleans, LA USA; 11 Cancer Service Line, Anne Arundel Medical Center, Luminis Health, DeCesaris Cancer Institute, Annapolis, MD USA; 12 Louisiana Cancer Research Center, Biospecimen Core, New Orleans, LA USA; 13 Department of Pathology, Tulane University School of Medicine, New Orleans, LA USA; 14 Stanley S. Scott Cancer Center, Louisiana State University Health Sciences Center, New Orleans, LA USA; 15 Department of Microbiology, Immunology, and Genetics, University of North Texas Health Science Center, Fort Worth, TX USA

**Keywords:** Triple negative breast cancer, Patient-derived xenograft, Extracellular matrix, Collagen, Metaplastic breast carcinoma

## Abstract

Metaplastic breast carcinoma (MBC) is a rare breast cancer subtype with rapid growth, high rates of metastasis, recurrence and drug resistance, and diverse molecular and histological heterogeneity. Patient-derived xenografts (PDXs) provide a translational tool and physiologically relevant system to evaluate tumor biology of rare subtypes. Here, we provide an in-depth comprehensive characterization of a new PDX model for MBC, TU-BcX-4IC. TU-BcX-4IC is a clinically aggressive tumor exhibiting rapid growth in vivo, spontaneous metastases, and elevated levels of cell-free DNA and circulating tumor cell DNA. Relative chemosensitivity of primary cells derived from TU-BcX-4IC was performed using the National Cancer Institute (NCI) oncology drug set, crystal violet staining, and cytotoxic live/dead immunofluorescence stains in adherent and organoid culture conditions. We employed novel spheroid/organoid incubation methods (Pu·MA system) to demonstrate that TU-BcX-4IC is resistant to paclitaxel. An innovative physiologically relevant system using human adipose tissue was used to evaluate presence of cancer stem cell-like populations ex vivo*.* Tissue decellularization, cryogenic-scanning electron microscopy imaging and rheometry revealed consistent matrix architecture and stiffness were consistent despite serial transplantation. Matrix-associated gene pathways were essentially unchanged with serial passages, as determined by qPCR and RNA sequencing, suggesting utility of decellularized PDXs for in vitro screens. We determined type V collagen to be present throughout all serial passage of TU-BcX-4IC tumor, suggesting it is required for tumor maintenance and is a potential viable target for MBC. In this study we introduce an innovative and translational model system to study cell–matrix interactions in rare cancer types using higher passage PDX tissue.

**Supplementary Information:**

The online version contains supplementary material available at 10.1007/s12094-021-02677-8.

## Introduction

Breast cancer remains the most common cancer affecting women, with 271,270 new cases in 2019 in the United States, with 30% of all new cancer diagnoses in women due to breast cancer [1]. In particular, the triple-negative breast cancer (TNBC) subtype represents 10–12% [2–4] of all breast cancers and is defined by the lack of estrogen receptor (ER), progesterone receptor (PR) expression, and human epidermal growth factor receptor 2 ((HER2) amplification. Compared to other breast cancer subtypes, TNBC is more aggressive clinically, has a worse prognosis, higher rates of metastasis to the brain and lungs [5, 6], higher histologic tumor grades and larger tumor volumes at initial clinical presentation [3, 7]. While most TNBC tumors are responsive to neoadjuvant chemotherapy, patients with unresponsive or poorly responsive tumors have overall worse prognoses and higher rates of relapse compared to other breast cancer subtypes [8–11]. Additionally, TNBC has a higher incidence in young, pre-menopausal Black and Hispanic women [6, 8, 12]. Metaplastic breast cancer (MBC) is a relatively rare and heterogeneous subtype of TNBC, comprising less than 1% of all breast carcinomas [13]. It is further characterized by neoplastic epithelial differentiation into squamous or mesenchymal-type tissue, resulting in the diverse presence of multiple cell types within the same tumor [14]. The World Health Organization identifies MBC as a heterogeneous group of breast cancers usually presenting as adenocarcinoma with dense areas of spindle cell, squamous, and mesenchymal differentiation [15]. Clinically, patients with MBC present with a rapidly growing palpable breast mass. Compared to a histologically different breast cancer type, such as infiltrating ductal carcinoma, MBC patients are older in age, more likely to be Black or Hispanic, less likely to have axillary nodal involvement, and more likely to present with larger tumor size and higher grade [16]. Patients with MBC have a lower overall 5-year survival when compared to invasive ductal or lobular carcinomas [17].

The intratumoral and molecular heterogeneity of TNBC, and specifically MBC, poses a distinct challenge in identifying effective therapeutic targets. Furthermore, due to the rarity of this disease, much of MBC biology remains widely unknown, especially with regards to the contribution of the ECM to oncogenesis and metastatic progression [22]. Most synthetic in vitro models utilized to mimic the native tumor microenvironment lack necessary components and results in an incomplete representation of the human tumor and its complex tumor microenvironment (TME) heterogeneity. The utilization of patient-derived xenograft (PDX) models have provided extremely valuable translational tools for therapeutic discovery research by addressing the heterogeneity of TNBC. Recent studies utilizing TNBC PDX models have characterized the wide range of tumor cell heterogeneity found within a single sample of TNBC [18, 19]. Additionally, PDX models can be utilized to characterize tumor evolution and metastasis, ultimately better able to predict chemotherapeutic responsiveness [20]. Of note, PDX explants and primary cell lines derived from MBC exhibit mixed tumor cell populations that are representative of the originally derived human tumor. Due to the complexity and heterogeneity of MBC cells, PDX models provide for an ideal, and highly translational, system for comprehensively understanding MBC biology. However, one of the limitations of utilizing PDX models in cancer research is that inevitably, after serial transplantation in murine models, mouse stromal components replace the human stroma [23, 24]. We hypothesized that the tumor matrix architecture and fiber alignment is relatively unchanged after serial transplantation, and we employed this concept to generate novel in vitro translational models using tissue decellularization to study the biology of rare tumor subtypes using higher passage PDX models.

Additionally, with serial transplantation in murine models, the relative amount of mouse stromal infiltration varies greatly amongst PDXs and is stable across serial passaging [24]. While the relative amount of mouse infiltration has been extensively examined, the effects of the mouse stromal composition on PDX tumor growth in murine models is lacking. Understanding genes and matrix components altered due to microenvironment adaptation may uncover underlying mechanisms of how tumors adapt to foreign environments. Thus, it will be important to parse out cellular processes and pathways necessary for tumor growth, as well as which pathways are duplicitous or expendable for MBC development.

There is an unmet need to identify viable biologic targets for this heterogeneous rare tumor subtype. In this proof-of-concept study we used a new translational PDX model and demonstrate that highly innovative methods can be utilized to interrogate unique features in rare tumors and that can be pursued as novel targets. Here we provide a comprehensive in-depth characterization of a new PDX model for MBC that we demonstrate can be utilized to study cell–matrix and matrix–matrix connections in higher passage models and, due to the rapid tumor take rates in vivo, this PDX model can be easily shared and used by various groups without waiting for an extended time for the tumor to grow in murine models.

## Methods

### Reagents

MDA-MB-231 cells were obtained from American Type Culture Collection (ATCC). Cells were grown in Dulbecco’s modified Eagle’s medium (DMEM; Caisson Laboratories, Smithfield UT) supplemented with 10% FBS (Gemini Bio-Products, West Sacramento CA), insulin, non-essential amino acids (NEAA, Caisson Laboratories), minimal essential amino acids, antibiotics and antimycotics, and sodium pyruvate (Invitrogen, Carlsbad CA) at 37 °C in 5% CO_2_. Dimethyl sulfoxide (DMSO) was purchased from Thermo Fischer Scientific.

### Patient-derived xenografts

TU-BcX-4IC was derived from a mastectomy specimen from a 57-years-old Caucasian female patient with MBC that was unresponsive to neoadjuvant chemotherapy with combination Adriamycin and cyclophosphamide therapy. TU-BcX-4IC was initially propagated in severe combined immunodeficient (SCID)/Beige mice (CB17.cg-*Prkdc*^scid^*Lyst*^bg^/Crl), purchased from Charles River that were 4–6 weeks old weighing approximately 10–18 g. Upon receipt of the freshly procured tumor specimen, we sectioned the tumor 5 × 5 mm pieces under sterile conditions and coated in a layer of Matrigel™ (Cat No. 354234, Corning Life Sciences, Corning, NY, USA) to improve the possibility of engraftment within the mouse. TU-BcX-4IC was implanted bilaterally into the mammary fat pads (mfp) of female SCID/Beige mice under general anesthesia, with a mixture of isoflurane and oxygen. Tumors were measured biweekly using digital calipers. Once the tumor volume reached 1000 mm^3^, TU-BcX-4IC was harvested and serially passaged into one new mouse. To clarify, each serial transplantation resulted in the transfer of a dissected PDX tumor piece into a new mouse. For example, with a total of eight serial passages of the transplanted tumor, nine total mice were utilized (including the mouse implanted with the original patient tumor). To passage PDX tumors, mice were euthanized with CO_2_ followed by cervical dislocation. Mice were given subcutaneous injections of melocixam (5 mg/kg/day) before surgery. Tumors were removed, dissected into 5 × 5 mm pieces, coated with Matrigel™ and transplanted bilaterally into another female SCID/Beige mouse under isoflurane anesthesia with oxygen. The nomenclature and labeling for serial transplantation of our PDX models in mice is denoted as Tx (with ‘x’ representing serial transplant number). For example, T0 represents the original PDX tumor (no exposure to murine models), T1 is the first serial transplant in a mouse, T2 is after two serial transplants, etc.

To calculate the rate of tumor growth, we used the following equation:

$${\text{Tumor}}\;{\text{Growth Rate}}\, = \,\Delta {\text{ tumor volume between measurements}}\, \div \,\Delta {\text{ days between measurements}}$$

### Digestion of tumor tissue

Treated tumor explants were harvested after 72 h. Explants were enzymatically digested in 100 µL Qiazol Lysis Reagent™ and minced using scissors. Tissue mixture was left for 30 min to completely homogenize the tissue. Samples were resuspended in additional Qiazol™. For phase separation chloroform was added, and samples were centrifuged at 14,000 rpm for 15 min at 4 °C. The top aqueous layer was transferred into a new microfuge tube and 100% ethanol was then added.

### Semi-quantitative real time PCR

Total RNA was extracted from digested 4IC tumor samples and from cell cultures with adherent TU-BCx-4IC cells using Quick-RNA MiniPrepTM (Zymo Research, Irvine, CA) according to the manufacture protocol. Quality and quantity of RNA were determined by absorbance at 260 and 280 nm. Then, RNA was reverse-transcribed (2 µg) into cDNA (iScript kit, BioRad Laboratories, Hercules, CA) and analyzed by RT-PCR. All RT-PCR data were normalized to actin. Primer sequences are as follows (Invitrogen, Carlsbad, CA): β-actin F-5’- GGCACCCAGCACAATGAAGA-3’; β-actin R-5’- ACTCCTGCTTGCTGATCCAC -3’; COL3A1 F-5’-TGCCCTACTGGTCCTCAGAA-3’; COL3A1 R-5’-TGCGAGTCCTCCTACTGCTA-3’; COL1A1 F-5’-CAGCCGCTTCACCTACAG-3’; COL1A1 R-5’-TTTTGTATTCAATCACTGTCTTGCC-3’; COL1A2 F-5’-CATTAGGGGTCACAATGGTC-3’; COL1A2 R-5’-TGGAGTTCCATTTTCACCAG-3’. Mouse-specific primers are: ms-COL1A1 F-5’- GAACTGGACTGTCCCAACCC-3’, R-5’- TCCCTCGACTCCTACATCTTCT-3’; ms-COL1A2 F-5’-CTAGCCAACCGTGCTTCTCA-3’, R-5’-TCTCCTCATCCAGGTACGCA-3’. RT-PCR analyses was performed using a real time PCR detection system (BioRad, Hercules, CA, USA) and a SYBR Green qPCR supermix kit (BioRad, Hercules, CA. USA) as per manufacturer’s protocol. Data were analyzed with a normalized gene expression method (ΔΔCt) using and the housekeeping gene β-actin was used for normalization. Data were represented as normalized fold expression compared with a DMSO control of biological triplicate samples ± SEM.

### NCI oncology drug set

TU-BcX-4IC primary cell line was established from the 4IC PDX tumor and maintained in DMEM supplemented with 10% FBS, insulin, non-essential amino acids (NEAA), minimal essential amino acids, antibiotic–antimycotic, and sodium pyruvate at 37˚C in 5% CO_2_. The established TNBC cell line MDA-MB-231, was utilized to compare relative drug responses. MDA-MB-231 and TU-BcX-4IC cells were seeded in 96-well plates and treated with the commercially available NCI oncology drug sets 4845 and 4846 (https://wiki.nci.nih.gov/display/NCIDTPdata/Compound+Sets). After three days, cells were fixed with glutaraldehyde and stained with Crystal Violet to observe response to chemotherapies. Representative images were captured with brightfield microscopy. Then, cells were then lysed with acetic acid (33%) and absorbance was measured at 620 nm wavelength with a spectrophotometer to indirectly measure the responsiveness to the chemotherapy.

### Quantification of cfDNA and CTC populations in blood

Mouse blood was collected in a tube containing EDTA and mixed by inversion. Aliquots of 100 µL blood were centrifuged at 2500×*g* for 10 min at room temperature (RT; 15–25 °C). Following the initial centrifugation step, the supernatant (plasma) was recovered and transferred into a 0.2 mL PCR tube. The cell pellet was kept for subsequent processing. The plasma was centrifuged at 16000×*g* for 10 min at RT to remove cells. The plasma was transferred into a new 1.5 mL low adhesion microcentrifuge tube and stored at – 80 °C. The two cell pellets from the centrifugation steps were combined and resuspended in remaining plasma, then washed twice with 150 µL of 1X PBS buffer (Growcells) to remove the leftover plasma and potentially contaminating cfDNA/ctDNA. The cell pellets were gently resuspended with the PBS buffer by mixing several times and centrifuged at 3300×*g* for 10 min at room temperature. The top layer of liquid was removed without disturbing the cell pellet. These steps for washing were repeated one more time.

cfDNA was extracted from 15 to 50 µL of the plasma with Quick-cfDNA Serum & Plasma Kit (Zymo-Research). Duplicate extractions were performed for each sample. The starting plasma volume and the final elution volume were recorded. Cellular DNA was extracted in duplicate from the cell pellet obtained from 100 µL of whole blood with Quick-cfDNA Serum & Plasma Kit (Zymo-Research). The protocol for “Purification of total (cellular and cell-free) DNA from Saliva” was followed with a single modification of doubling the proteinase K digestion time. The cell pellet was first resuspended with nuclease free water (VWR) to make the volume approximately 200 µL. The extracted DNA was eluted with 100 µL of nuclease free water, and the recovered volume was recorded. Quantitative real time PCR assays were performed with Brilliant Multiplex QPCR Master Mix (Agilent Technologies) on the Applied Biosystems 7500 Real Time PCR instrument. The three-target primer mix contains three primer–probe sets that amplify simultaneously a 80 bp DNA region targeting human-specific Alu Yb8 retrotransposons, 90 bp DNA region targeting mouse beta-2 microglobulin gene, and synthetic 172-base DNA oligo that serves as an internal PCR control (IPC). The inclusion of the synthetic IPC in each amplification provides a measurement for the presence of any PCR inhibitors in the sample or the failure of PCR reaction by any pipetting error. The extracted DNA samples were amplified in duplicate in a 20 µL reaction volume. The DNA sample volumes for qPCR reactions were 4 µL for the ctDNA detection and 2 µL for the CTC-DNA detection. A freshly prepared eightfold serial dilution of high molecular weight standard human DNA (2.5 ng/µL, 0.3125 ng/µL, 0.039 ng/µL, 0.005 ng/µL, and 0.0006 ng/µL) and fourfold serial dilution of mouse standard DNA (10 ng/µL, 2.5 ng/µL, 0.625 ng/µL, 0.156 ng/µL, and 0.039 ng/µL) were run in duplicate to generate the standard curve. The PCR conditions involved a single enzyme activation cycle for 10 min at 95 °C, followed by 45 cycles of 2-step qPCR (15 s at 95 °C and 2 min at 61 °C combined annealing/extension time) at maximum ramp speed. To correct for well-to-well variations in background fluorescence on the 7500, the ROX-labeled passive reference dye was used. The resultant quantification results were then normalized using the following equations to reflect ctDNA/CTC-DNA conditions in mouse blood.

***Equation 1***. Conversion of absolute ctDNA/cfDNA concentration in plasma from quantification of extracted DNA.

$$\begin{aligned}& {\text{Absolute ctDNA}}/{\text{cfDNA concentration in plasma }}\left( {{\text{pg}}/\mu {\text{L}}} \right)\\&\quad = \,{\text{qPCR measured concentration }}\left( {{\text{ng}}/\mu {\text{L}}} \right)\\&\qquad \times \,{\text{extraction elution volume }}\left( {\mu {\text{L}}} \right)\\&\qquad \div \,{\text{extraction volume of plasma }}\left( {\mu {\text{L}}} \right)\, \times \,{\text{1}}000. \end{aligned}$$

***Equation 2***. Conversion of absolute CTC DNA/mouse leukocyte DNA concentration in blood from quantification of extracted DNA.

$$\begin{aligned} &{\text{Absolute human CTC DNA and mouse leukocyte }}\\&{\text{DNA concentration in blood }}\left( {{\text{pg}}/\mu {\text{L}}} \right)\\&\quad = \,{\text{qPCR measured concentration }}\left( {{\text{ng}}/\mu {\text{L}}} \right)\\&\qquad \times \,{\text{extraction elution volume }}\left( {\mu {\text{L}}} \right)\\&\qquad \div \,{\text{extraction volume of blood }}\left( {\mu {\text{L}}} \right). \end{aligned}$$

### Immunohistochemistry staining

Briefly, tumor specimens from each passage, lungs, and livers were fixed in formalin, then embedded in paraffin. Formalin-fixed, paraffin-embedded specimens were stained with Hematoxylin and Eosin (H & E) and imaged at 20X objective using an Aperio Scanscope instrument (Aperio Technologies, Inc., Vista, CA, USA). Images of the lungs and liver were analyzed for evidence of metastasis using ImageScope software (Aperio Technologies, Inc.)

### Flow cytometry and fluorescence activated cell sorting (FACS)

To analyze CSC phenotypes, TU-BcX-4IC was enzymatically digested with type I collagenase (Worthington Biochemical Corporation, Lakewood, NJ, USA) at room temperature, neutralized with media, and then filtered. Circulating tumor cells were collected in whole blood with 0.5 M EDTA (Gibco Invitrogen, Carlsbad CA), incubated in red blood cell lysis buffer (0.008% NH_4_Cl, pH 7.2–7.4; Sigma-Aldrich, St. Louis MO) and washed with PBS. Collected cells from the tumor and blood samples were placed in a staining solution containing 1% Bovine Serum Albumin (BSA; Sigma-Aldrich) and 1% CD16/CD32 Mouse BD Fc Block™ (BD Biosciences) in PBS. The following primary antibodies were used: Anti-human CD24 (APC) and anti-human/mouse CD44 (PE-Fluor 610) purchased from eBiosciences (San Diego, CA, USA). All cells from the blood were analyzed with a Galios Flow Cytometer (Beckman Coulter, Brea, CA, USA) running Kaluza software (Beckman Coulter). At least 5,000 events were analyzed and reported as the mean ± SEM.

### Live/dead fluorescent stain for adherent TU-BcX-4IC cells

TU-BcX-4IC cells were treated with DMSO or paclitaxel (10 nM) for 72 h. Media was removed and cells were stained using the PromoKine live/dead staining kit (New York, USA). Cells were exposed to Calcein-AM (2 µM) and Ethidium homodimer (EthD)-III (5 µM) mixed with phosphate buffered saline. Calcein-AM can be transported through the cell membrane of live cells, where fluorescence activation is based on interaction with esterase enzymes. Ethidium homodimer binds to DNA of lysed (dead) cells. Cells were incubated for 45 min. Stained cells were imaged with confocal fluorescence microscopy and images were captured (8 images per well of adherent cells, 5 images per well of low-suspension cells). The 588 nM excitation channel was used to identify red, ‘dead’ cells, and the 420 nM excitation channel was used to visualize green, ‘live’ cells. Representative images were taken at 100× magnification.

### Automated flowchip assay

The Pu·MA system device (Protein Fluidics, Burlingame CA) comprises a bench-top instrument, that easily fits into a standard tissue culture incubator, containing microprocessor controlled pneumatic system, a touchscreen interface, and a plate holding chamber [25]. A key component of the automation system is the Pu·MA System flowchip. The flowchips are designed with chambers and wells in a convenient multi-well plate format (SLAS/ANSI 384-well plate standard) that are connected by microfluidic channels. The flowchips are made from black cyclic olefin copolymer (COC) with a thin, optically clear COC bottom suitable for multiple assay read-outs including high resolution imaging. Reagents (20 µL; media containing treatment groups; stains) and spheroids were loaded into the device manually or using any automated 384-well compatible liquid dispense system. Spheroids were formed externally and then placed into a sample well along with 20 µL of buffer or media. Spheroids or organoids were positioned into a special protected chamber at the bottom of the sample well. After sample loading, the plate was placed into the Pu·MA System and reagent exchanges were done automatically through the microfluidic channels. The Pu·MA System was placed in an incubator to run assays at 37 °C and 5% CO_2_. The system architecture and use of pneumatics moved fluids provides gas exchange to the sample chambers. The underlying technology of the microfluidic-device is Valve-less Fluidic Switching (VLFS). Hydrophobic barriers at entrances and exits of the channels keep fluids in place until pneumatic pressure differentials are applied to move the fluids from well-to-well. An on-board microprocessor-based controller provides instructions to the pressure control system.

### TU-BcX-4IC organoid models

Organoids were maintained as described above. For the concentration response studies and phenotypic assay, cells were cultured in ultra-low attachment plates in the appropriate media at 37 °C and 5% CO_2_. Spheroids were formed over a 2- to 3-day period and then transferred into flowchips for compound treatment and staining. For compound effects, compounds (Sigma-Aldrich) were typically tested in duplicates in a six-point dilution series. Spheroids were transferred into flowchips and treated by performing an automated media exchange. Cells were then exposed to the various concentrations of compounds for 48 h.

### Multiparametric live cell toxicity assay

The method for imaging and high content analysis of 3D spheroids was previously described [25, 26]. Briefly, following incubation with test compounds, spheroids were stained with a mixture of three dyes: 1 µM calcein AM, 3 µM of EthD-1, and 33 µM Hoechst 33,342 (Life Technologies, Carlsbad, CA). Spheroids were also stained with E-cadherin (24E10) (Cell Signaling Technology #3195, Danvers, MA) and CD44 (BioLegend #338,808, San Diego, CA). Automated staining of spheroids was performed for one hour. After staining, dye solution was replaced with 1X PBS as a wash step at the end of the process.

### High-content imaging

Images were acquired using confocal automatic imaging system, ImageXpress Micro Confocal (Molecular Devices, San Jose, CA), as previously described [27] with a 10X Plan Fluor objective or 20X Plan Apo. DAPI, FITC and Texas Red filter sets were used for imaging. A stack of 7–15 images separated by 10–15 µm was acquired, starting at the well bottom and covering approximately the lower half of each spheroid. Typically, a Z-stack of images covered 100–200 µm for spheroids. Image analysis was performed either in 3D or using the 2D Projection (maximum projection) images of confocal image stacks. Transmitted light images were used for cell culture monitoring or protocol optimization.

### Organoid image analysis

Images were analyzed using MetaXpress High-Content Image Acquisition and Analysis Software (Molecular Devices). Count Nuclei or and Cell Scoring application modules were used for nuclear count live/dead assessment, or evaluation of cell number positive for specific markers. Output measurements included spheroid width, spheroid area, average intensity for calcein AM or EthD-1, counts of all nuclei, and evaluation of average nuclear size and average intensities. In addition, calcein AM positive cells were counted, and their area and intensity values were recorded. In addition to cell count, areas and intensities could be determined for Live (EthD-1 negative) and Dead (EthD-1 positive) cells. EC50 values were determined using 4-parameter curve fit from SoftMax Pro 6 software (Molecular Devices) [26].

### Cryogenic scanning electron microscopy and transmission electron microscopy

Frozen tissue samples were decellularized and analyzed by cryogenic-scanning electron microscopy. PDX tumors were flash frozen and later thawed for 30–45 min followed by sectioning using an 8-mm biopsy punch (Catalog No. P825; Acuderm Inc., Ft. Lauderdale, FL, USA). Sectioned PDXs were decellularized through a protocol modified from Pashos et. al. [28]. Samples were incubated overnight with PBS at 4° and then washed with water for 2 h the following day. Next, samples were incubated with a Triton X-100 detergent solution followed by a 2 h water wash. Then, the samples were incubated with a sodium deoxycholate solution (Catalog No. 97062–024; Amresco, Solon, OH, USA) followed by a 2 h water wash. Finally, samples were incubated with a sodium chloride solution for 2 h followed by a 2 h water wash. Samples were stored at 4 °C in a PBS solution containing 5 × antibiotic/antimycotic until use. The extracellularized matrix of decellularized PDXs were imaged using scanning electron microscopy (SEM). Samples were fixed in FAA (formaldehyde-acetic acid–ethanol) fixative for 24 h. The samples were rinsed and dehydrated with an increasing series of ethanol, from concentrations 30% to 100%. After the dehydration process, the samples were dried with CO_2_ at a previously defined ‘critical point’ and then sputter coated (lightly coated) with platinum. The prepared SEM samples were then imaged using a FEI Quanta 3D FEG FIG/SEM scanning electron microscope (5 kV).

### Next generation sequencing of TU-BcX-4IC tumors

TU-BcX-4IC tumors (T0, T4) were extracted for total RNA and samples were analyzed using next-generation sequencing techniques. The following were removed from analysis: small non-coding-lincRNA, miRNA transcripts, 3-prime overlapping ncRNA, processed pseudogene and anti-sense RNA. Only protein coding RNA transcripts were retained and analyzed. Data was aligned to human genome and mouse genomes. ‘Unique genes’ were the genes only aligned to the human genome and did not include genes that mapped to both human and mouse genomes based on similar genetic sequences. Fold changes were identified as increased if gene fold change compared to parental was above 1.5, and gene fold changes were identified as decreased if below 0.5-fold change. All genes were corrected for false discovery rate (FDR) and were considered significant if adjusted p-value (FDR) was < 0.05. Identification of the most significantly altered pathways was performed through David’s Bioinformatics Resources v6.8 (https://david.ncifcrf.gov/home.jsp). Gene changes for associated pathways were identified through the gene ontology database. Data shown represents fold change in the original PDX tumor (T0) compared to lower passage (T1) and higher passage (T4) serially transplanted tumors.

### Statistical analyses

Studies that were performed in triplicate were analyzed by unpaired Student’s *t-*test (Graph Pad Prism V.4). p-values of p < 0.05 were statistically significant. The flow cytometry analysis of the circulating tumor cells, detection of liquid biopsy markers, and qPCR analyses of tumors were performed in duplicate.

## Results

### TU-BCx-4IC exhibits rapid tumor cell adhesion and growth in vivo while maintaining malignant features in serial transplantation in mice

**Fig. 1:**
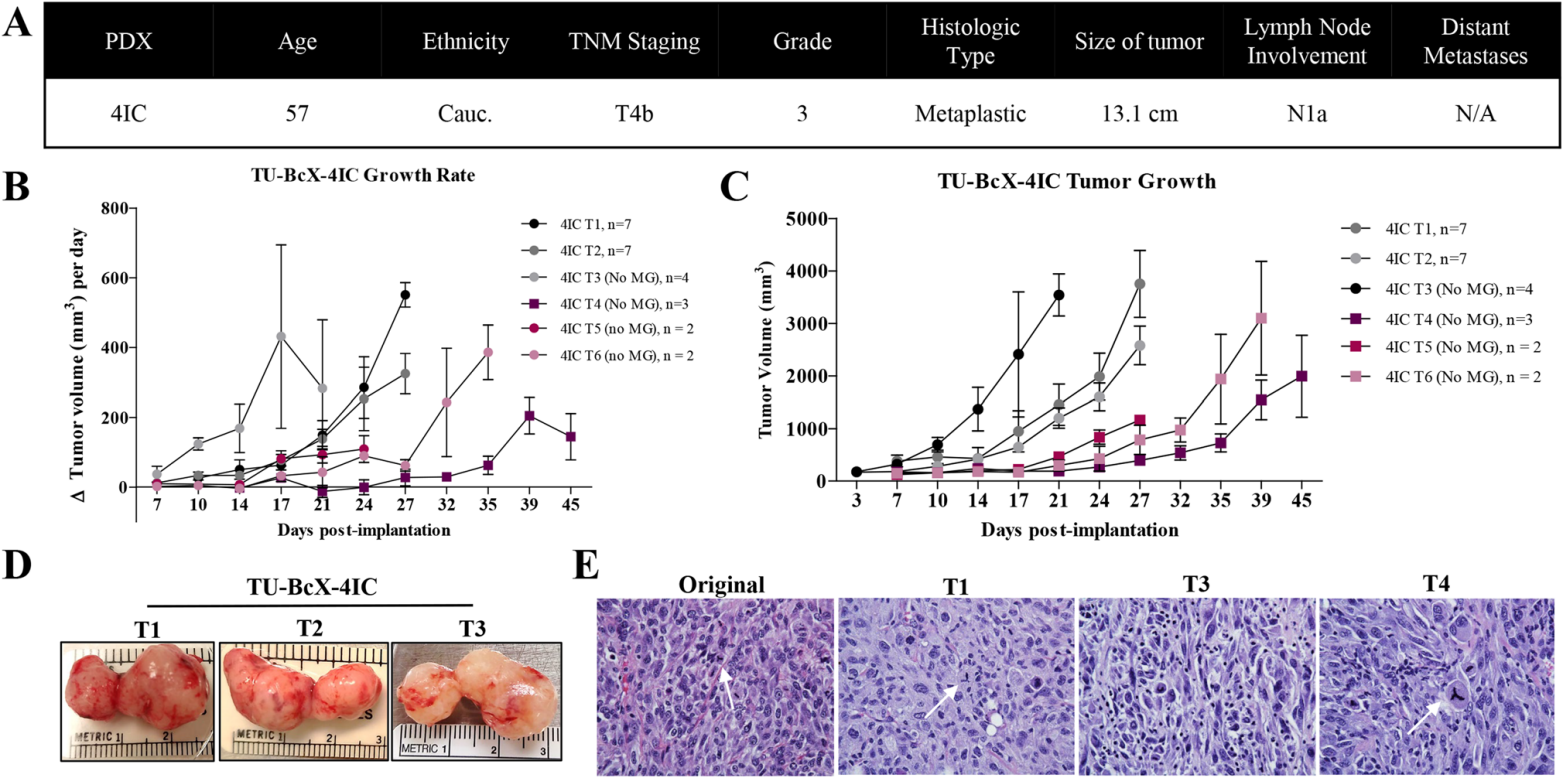
Establishment and characterization of TU-BcX-4IC. **A** Information about the patient from which TU-BcX-4IC was derived. The tumor was characterized as a TNBC subtype and classified as a metaplastic breast carcinoma. **B** TU-BcX-4IC immediately grew after transplantation, a feature that was consistent in serial transplantation. After 5 passages, the latency period before achieving maximal tumor volume was prolonged. MG = Matrigel. **C** The observed initial tumor growth rate varies in early- and late-passage PDX models, with slower growth rates seen in higher passages. The growth rate was calculated as the difference in tumor volume over the biweekly measurement intervals. **D** TU-BcX-4IC after serial transplantation in SCID/Beige mice. **E** Representative images of Hematoxylin and Eosin (H&E) stained TU-BcX-4IC tumors from serial passages in mice. Early- and late-passage PDX retain the aberrant mitotic figures observed in the primary human tumor specimen, as represented by white arrows in the images

The primary tumor from which TU-BcX-4IC was derived exhibited rapid pre-operative growth despite combination neoadjuvant therapy with adriamycin, cyclophosphamide and paclitaxel. At surgery, the patient presented with multinodular skin lesions with significant ulceration at the lateral margin and invasion of the pectoralis muscle. TU-BcX-4IC was classified as MBC with a TNBC subtype, lesion involvement of all four sampled quadrants of the breast, along with cystic degeneration and necrosis (Fig. 1A). The tumor sample implanted in SCID/Beige mice exhibited rapid tumor growth, with 14 days to maximal tumor volume > 1000mm^3^, compared to the mean length of time to the maximum volume of other established TNBC PDX models [23, 24, 27]. Serial transplantation in mice is denoted as Tx (with ‘x’ representing serial transplant number, for example, T1 is the first transplant in mice). Importantly, we observed that while early passage tumors were coated with Matrigel™ per our implantation protocols to establish new PDX models, after T2 Matrigel™ was not required for growth. All serial transplants successfully grew with consistently rapid growth rates in the absence of Matrigel™ (Fig. 1B). Early passages of TU-BcX-4IC in mice had short latency periods, defined as the time from implantation to initial tumor formation, with early passage tumors having faster growth rates when compared to higher passage tumors (Fig. 1C). TU-BcX-4IC exhibited a consistently homogenous and highly cellular solid tumor specimen upon microdissection with all serial transplants (Fig. 1D). TU-BCx-4IC cell histology was consistent in both lower and higher passages. Representative images of H & E-stained TU-BCx-4IC tumors revealed the cellular composition of serial passages were heterogeneous, with tumor cells exhibiting aberrant mitoses, marked hyperchromatism, and surrounding fibrosis. Notably, tripolar mitotic figures were consistently present in both lower and higher passages (Fig. 1E).

### TU-BcX-4IC spontaneously metastasizes and exhibits a prominent CSC population

**Fig. 2:**
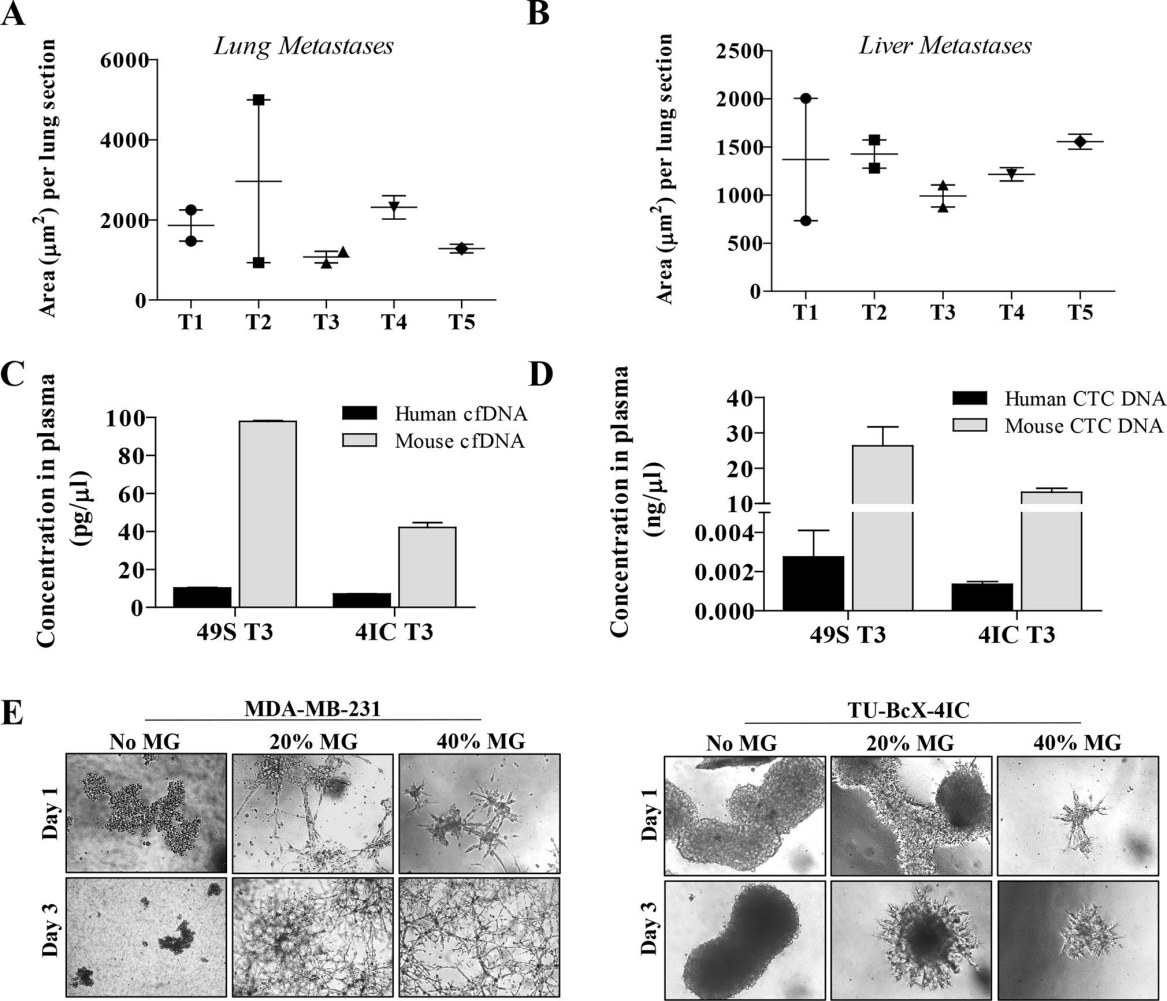
TU-BcX-4IC cells have an invasive, metastatic phenotype and are resistant to oncology drugs in vitro. Lungs and livers were harvested after serial passaging of TU-BCx-4IC in SCID/Beige mice (passages 1, 3 and 5 denoted T1, T3, T5). Organs were fixed, paraffin embedded, sectioned and stained with H & E to observe metastases. Quantification of lung metastases showed that area **A** of metastases per lung section were consistent over serial transplantation. Quantification of liver metastases showed that area **B** of metastases per liver section were also consistent over serial transplantation. **C** Peripheral blood was harvested from T3 passage of TU-BcX-49S and TU-BcX-4IC models. Human and mouse circulating cfDNA were detected in both PDX models. Although there was no difference between human cfDNA in the models, TU-BcX-49S had higher measurable levels of mouse-specific cfDNA. **D** TU-BcX-49S also exhibited higher levels of human circulating tumor cells compared to TU-BcX-4IC. Mouse DNA was utilized as the housekeeping gene in these experiments. **E** TU-BcX-4IC and MDA-MB-231 cells were embedded in Matrigel (0%, 20%, 40%) in 3D culture conditions. Representative images of embedded spheres were recorded after one and 3 days

TU-BcX-4IC formed spontaneous metastatic lesions, consistent with the aggressive clinicopathologic behavior of MBC (Supplementary Fig. S1). The average area of metastases per lung and liver sections was consistent throughout all serial passages in mice (Fig. 2A, B) and the average number of metastatic lesions per lung and liver sections were increased in higher passage mice (Supplementary Figure S2). The slight discrepancies in area and number of metastases in individual passages is partially due to the different growth rates of the associated tumors (Fig. 1B, C). On gross examination at the time of necropsy, metastases were observed in the lungs and liver in all passages in vivo.

To further characterize our new PDX tumor, we evaluated levels of liquid biopsy biomarkers, or cell-free DNA (cfDNA) and circulating tumor cell DNA (CTC DNA), which have been used to predict metastases in PDX models and patients [30, 31]. We compared relative cfDNA and CTC DNA within TU-BcX-4IC tumors to TU-BcX-49S, another metastatic TNBC model established in our laboratory. At necropsy, mice implanted with the same passage (T3) of TU-BcX-4IC and TU-BcX-49S PDX models had similar metastatic profiles (Supplementary Fig. S3), except liver metastases were more abundant in TU-BcX-4IC. Consistent with the metastatic potentials of these PDX models, levels of human and mouse cfDNA and human circulating tumor cell DNA (CTC DNA) were present in both TU-BcX-4IC and TU-BcX-49S tumors (Fig. 2C, D). Notably, TU-BcX-4IC had a faster growth rate than TU-BcX-49S (Supplementary Figure S4). We generated a primary cell line derived from the original mastectomy specimen of TU-BcX-4IC. When TU-BcX-4IC cells were seeded in Matrigel containing suspension culture conditions, they exhibited protrusions, features characteristic of migratory and invasive TNBC cell phenotypes, including MDA-MB-231 cells (Fig. 2E).

**Fig. 3:**
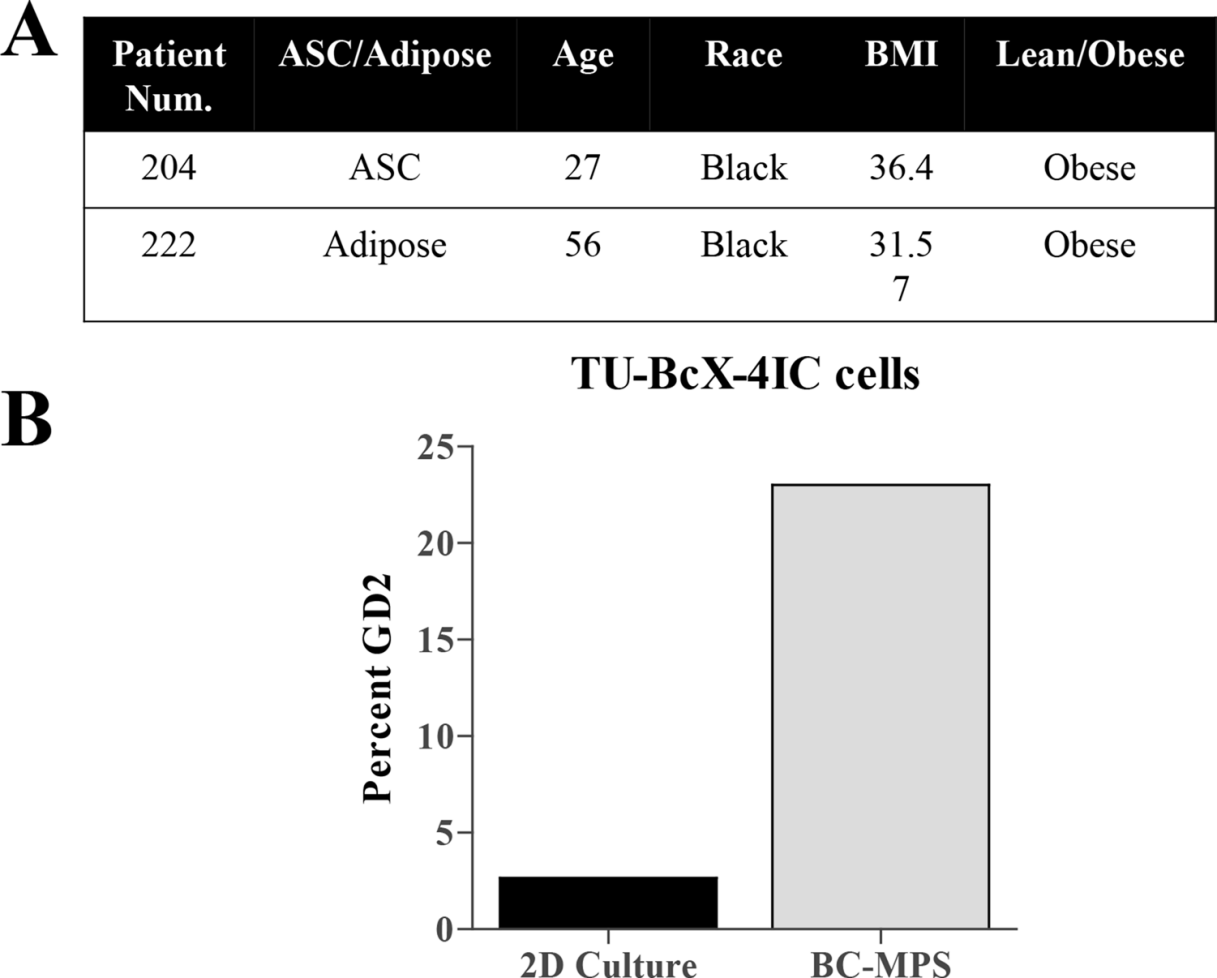
TU-BcX-4IC cells embedded in human adipose tissue maintain presence of CSC populations. CSC populations were evaluated in TU-BcX-4IC cells plated in vitro in a physiologically relevant microenvironment using human breast adipose tissue (the BC-MPS system). **A** Demographics of the patients from with the ASCs or adipose tissue was derived is shown. **B** Flow cytometry of the CSC population (% GD2 positivity) within TU-BcX-4IC cells revealed a fourfold increase when cultured in BC-MPS compared to standard 2D culture

The presence of a cancer stem cell-like population is associated with tumor recurrence, metastasis and overall more aggressive clinical and biological features [32–34]. TU-BcX-4IC tumors exhibited high populations of CSC-like cells, characterized as CD44^+^CD24^−^ (27.5% total cell population) or Ganglioside (GD2)^+^ [35] (44.8% total cell population) (Fig. 3A). To confirm cells derived from the PDX model maintained the CSC population in vitro, we employed a new translational system developed to provide physiologically relevant tumor microenvironment support to in vitro culture conditions, denoted the breast cancer microphysiological system (BC-MPS). In this system, modified from the system described in Lau et al. [36], human breast adipose tissue is plated with cancer cells in adherent or suspension conditions. For this project, the patient number was recorded to match patient demographics. The adipose-derived stem cells (ASCs) used in the BC-MPS in which the TU-BcX-4IC cell line was cultured was from an obese patient (204) with a BMI of 36.4 (Fig. 3B). The adipose tissue that was placed in the same BC-MPS was from a different obese patient (222) with a BMI of 31.7. There was approximately a fourfold increase in the cancer stem cell population when cultured in BC-MPS compared to standard adherent culture conditions (Fig. 3A, B; Supplementary Figure S5).

### TU-BcX-4IC-derived cells and organoids are resistant to paclitaxel

**Fig. 4:**
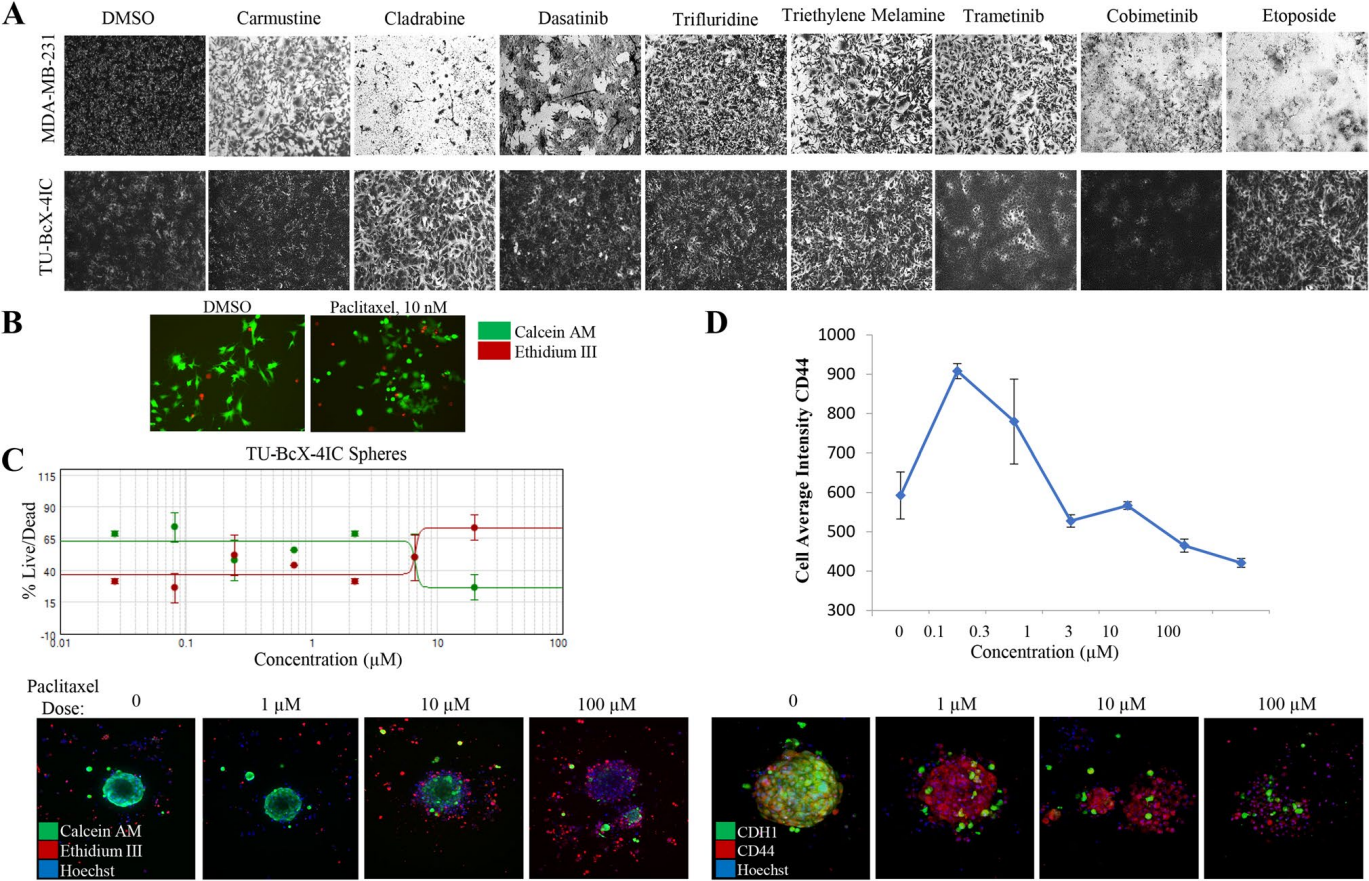
Chemosensitivity profiles of TU-BcX-4IC cells. **A** TU-BcX-4IC-derived cells and MDA-MB-231 cells were treated with the NCI-approved oncology drug panel for three days. Overall, TU-BcX-4IC cells were more chemoresistant than MDA-MB-231 cells. **B** TU-BcX-4IC adherent cells were resistant to paclitaxel treatment (10 nM, 72 h), observed with a live/dead Calcein-AM/EthD III fluorescent stain. Green = live cells, Red = dead cells. **C** U-BcX-4IC organoids were treated with varying doses of paclitaxel (1 µM, 10 µM, 100 µM) and stained with the live/dead cytotoxicity stain and Hoechst nuclear stain using the Pu·MA system (Protein Fluidics, Burlingame CA) and images were obtained using confocal microscopy (Molecular Devices, San Jose CA)

The original tumor was clinically aggressive, resistant to three cycles of adriamycin, cyclophosphamide and paclitaxel therapies. Our TU-BcX-4IC PDX model can be used as a model for groups studying taxane resistance; thus, we then sought to demonstrate that the cells and organoids derived from the PDX model reflected the poor response of the original tumor to taxane-based therapies a classic chemotherapy regimen. Relative drug sensitivity of TU-BcX-4IC cells to FDA-approved anticancer drugs compared to MDA-MB-231 cells was then evaluated using the National Cancer Institute drug set, treated at 1 µM. Overall, TU-BcX-4IC cells were less responsive with respect to cell survival (Fig. 4A). Cells were treated at 1 µM and relative effects on cell viability were evaluated using crystal violet staining. Compared to MDA-MB-231 cells, TU-BcX-4IC cells overall exhibited more resistance to commonly used systemic and targeted therapies. TU-BcX-4IC cells were completely resistant to some drugs, including alkylating agents (carmustine), anti-metabolites (cladribine), and topoisomerase inhibitors (etoposide) (Fig. 4A; Supplementary Fig. S6). TU-BcX-4IC cells were partially resistant to tenoposide, valrubicin, vinorelbine and vinblastine (Supplementary Figure S6, Supplementary Figure S7) and we also identified drugs that induced cytotoxic effects on TU-BcX-4IC cells at the 1 µM screening dose (Supplementary Figure S8). Due to the resistant nature of the original tumor to paclitaxel, we further investigated the response of TU-BcX-4IC adherent cells to this treatment; in a live/dead cytotoxicity immunofluorescence stain at physiologically relevant doses (10–100 nM) of paclitaxel TU-BcX-4IC cells did not exhibit cytotoxicity (Fig. 4B). Spheroids derived from TU-BcX-4IC were then treated with paclitaxel and stained using the innovative Pu·MA microfluidics transfer system and images were obtained using a novel immersion fluorescence imaging system. Paclitaxel caused cytotoxicity in TU-BcX-4IC spheroids and organoids, but only at high concentrations (Fig. 4C). Quantification of live/dead cytotoxicity stain with TU-BcX-4IC organoids treated with paclitaxel. EC50 range was determined from this quantification, with a range of 6.8–29.4 µM for spheroids and 6.5–24.9 µM for organoids (Fig. 4C, Supplementary Figure S9), values much higher than physiologically relevant doses. Furthermore, paclitaxel treatment led to reduced expression of the CSC marker CD44 and the epithelial marker E-cadherin (CDH1) expressions in a dose-dependent manner (Fig. 4D).

### RNA sequencing of TU-BcX-4IC tumors reveals differences in the gene expression landscape of tumors after serial transplantation in mice

RNA sequencing was utilized to evaluate overall changes to the genomic landscape due to serial transplantation of TU-BcX-4IC in SCID/Beige mice. We compared tumors from the original patient specimen (T0), tumors passaged once in a mouse (T1) and tumors that were serially transplanted four times in murine models (T4). To determine gene expression changes over serial passaging, RNA sequencing was aligned to both mouse and human genomes and only unique reads per gene per genome were evaluated. Overall, there were more genes uniquely mapped to the human genome in T4 (43% of the total number of genes analyzed) compared to T1 (27.5%) and T0 (23%) tumors. As expected, there were more uniquely mapped genes to the mouse genome in higher serial passage tumors (T4; 0.87% of the total number of genes analyzed) compared to lower passage tumors (T1 0.75%) (Table 1).

**Table 1:** Relative abundance of human-specific and mouse-specific genes present in lower passage (T1) and higher passage (T4) tumors compared to the original TU-BcX-4IC specimen (T0). Percentages are shown in relation to the total genome analyzed with RNA sequencing

	T0	T1	T4
Human specific	23%	27.5%	43%
Mouse specific	0.03%	0.75%	0.87%

**Fig. 5:**
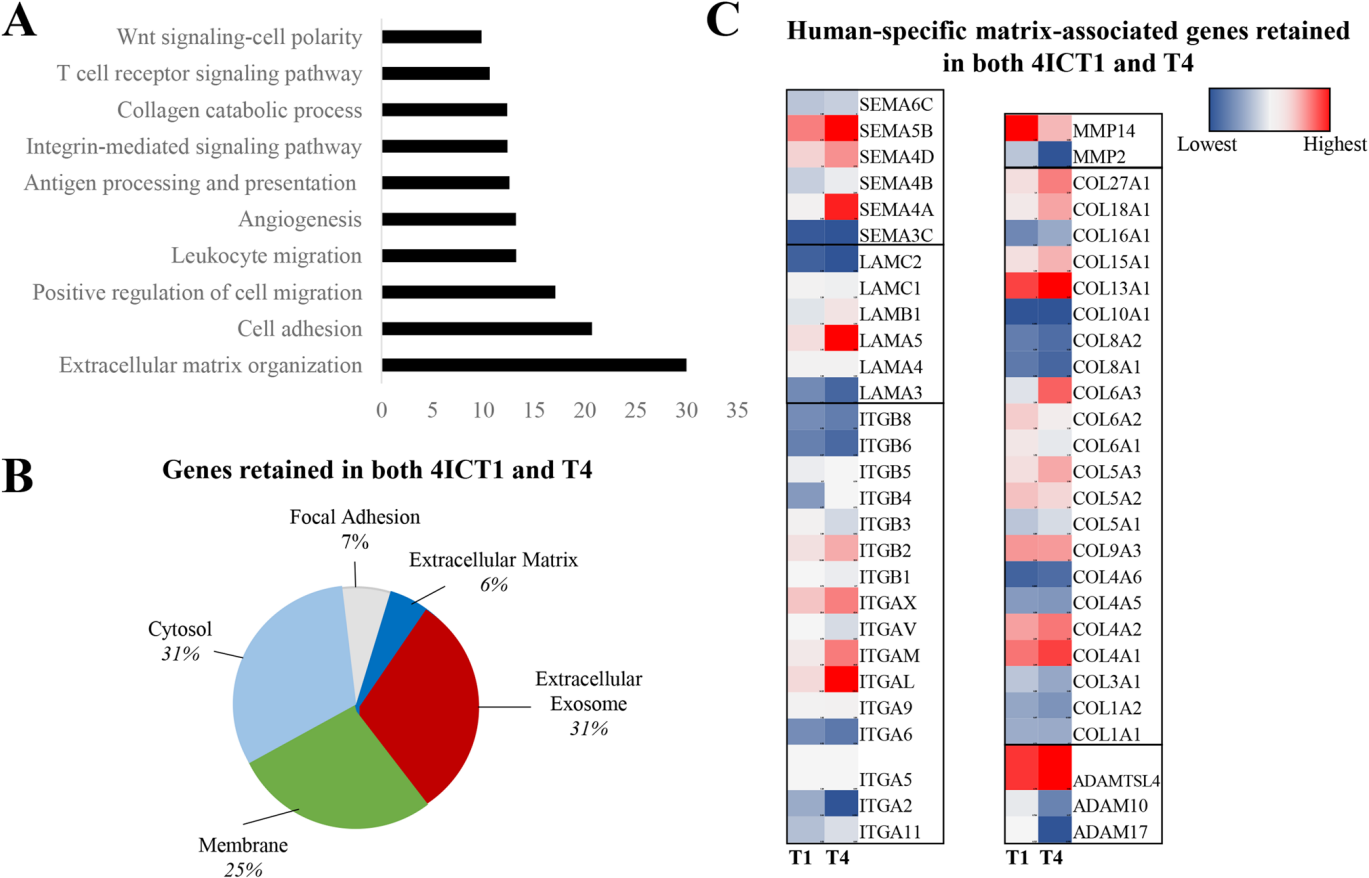
RNA sequencing of serial passages of TU-BcX-4IC compared to the original patient tumor. RNA sequencing was performed on TU-BcX-4IC tumors representing serially transplanted tumors in mice (T1, T4), compared to the primary tumor (T0). Data represents changes in genes unique to the human genome following removal of mouse aligned genes. **A** Top ten signaling pathways upregulated by both T1 and T4 tumors included ECM organization, cell adhesion and positive regulation of cell migration. **B** Representation of signaling pathways retained in both TU-BcX-4ICT1 and T4 tumors. **C** Genes involved in ECM organization, positive regulation of cell matrix, and angiogenesis that were upregulated in both TU-BcX-4ICT1 and T4 tumors. Up-regulated genes included those in collagen, integrin, laminin, metalloproteinase and the semaphorin gene families. Data is represented by a heat map for each gene listed, with blue as the lowest value within a gene group and red as the highest value. Each heat map scale for specific genes (T0, T1, T4 passages) are analyzed separately to represent changes in gene expression over serial transplantation. For example, the heat map range for the semaphorin or laminin gene clusters are unique from the other gene clusters, outlined by black boxes in the heat map

Gene ontology biological processes was used to examine specific cellular changes between T0 and the T1 and T4 tumors. Overall, pathways that were most upregulated in higher passage tumors, compared to T0 tumors, included those associated with the tumor microenvironment (extracellular matrix organization, cell adhesion, cell and leukocyte migration, angiogenesis and integrin/collagen signaling pathways) (Fig. 5A). These data suggest that the tumor microenvironment (TME) is remodeled over serial transplantation in mice. To better identify which components of the TME were being remodeled with serial transplantation, i.e. secreted factors, exosome associated factors, adhesion molecules, matrix fibers, we next performed gene ontology analysis for cellular component localization. The genes most consistent from T0 to T4 were localized in the extracellular exosome (31%) and cytosol (31%), the cell membrane (25%), genes involved in focal adhesion (7%) and the extracellular matrix (6%) (Fig. 5B). Then, specific genes retained in higher passages compared to lower passages within these pathways were examined (Table 2, Fig. 5C). Overall, the most prominent genes highlighted in this analysis were from matrix-associated gene families, including the integrins, laminins, collagens, semaphorins and metalloproteinases. Of note, the analysis suggested that many of the collagens were increased in serial transplantation are fiber-associated collagens (I, III) as well as collagens identified to be associated with triple-negative breast cancer (X). Fat-associated collagens (VI) were decreased, which may be indicative of the sequential loss of human adipose with serial transplantation, as were collagens associated with the basement membrane (IV). Together, this demonstrates that the TME and cellular adhesion mechanisms evolved during PDX serial transplantation in murine models on a transcriptomic level. Enrichment of TNBC associated collagens (collagen X) and repression of stromal-associated collagens (VI) suggest serial passage supports tumor matrix development.

**Table 2:** Specific genes involved in ECM organization, positive regulation of cell migration, and angiogenesis identified from RNA sequencing that were retained in both lower passage (T1) and higher passage (T4) tumors, compared to the original tumor (T0)

ECM Organization	Positive Regulation of Cell Migration	Angiogenesis
ADAMTSL4, ATP7A, CD44 CD47, SMOC2, TNFRSFllB, APP, B4GALT1, BGN, CCDC80, DCN, DAG1**, **ELN, FENl , FEN2, HSPG2, JAM2, KDR, LUM, LOXLl , NFl, NIDI , NID2, QLFML2B, POSTN, PXDN, PRDX4, SPPl , SPARC, SPINTl , SERPINBS, SERPINEl, THBSl, TGFBI, VCAMl, VCAN, VWF	APC, CCR, CXCL16, DAB2, HRAS, TNFAIP6, ACTN4, AIFl, BMP4, CREB3, CIBl , CARMILl, CTSH, CEMIP, F3, CSFIR, COROlA, DIAPHl , FERMT3, FLTl , GPNMB, HSPAS, HAS2, IGFlR, INSR, KDR, LGR6, MMP14, MCAM, MYADM, MYOlC, MYLK, NOTCHl , PIK3CD, PLAU, PDGFC, PDGFRB, PODXL, RACK I , RRAS2 , SERPINB3, SPAG9, SDCBP, THBSl , TGFBRl , VEGFA	ATPSB, CCL2, CXCL8, EPHB2, HTATIP2, NOXS, NUSl, ARHGAP22, SHCl , TNFRSF12A, THYl , ACVRLl , ANPEP, ANGPTL4, ANXA2, CIBl , CLIC4, CSPG4, DLL4, ERAPl , EPASl , EFNAl , ECMl , FLTl , FMNL3, FZD8, HMOXl , HSPG2, HEYl , HIFlA, KDR, MCAM, MFGE8, NOV, NRXN3, NRCAM, NRPl , TNC, NCL, PNPLA6, PTEN, PIK3CG, PLXNDl , SCG2, SERPINEl , SLC12A6, SY, TGFBl , TIEl , UNCSB, VEGFA VEGFC VASHl
COLlAl , COL1A2, COL3Al , COL4Al , COL4A2, COL4AS, COL4A6, COL9A3, COLSAl , COLSA2, COLSA3, COL6Al , COL6A2, COL6A3, COL8Al , COL16Al COL18Al COL27Al	COLlAl , COL18Al	COL4A2, COL8Al , COL8A2, COLlSAl , COL18Al ,
ITGAll , ITGA2, ITGA4, ITGAS, ITGAV, ITGAX, ITGBl , ITGB2, ITGAV ITGB3, ITGB4, ITGBS, ITGB6, ITGB8	ITGAS, ITGA6, ITGAV	ITGAS, ITGAV
LAMA3, LAMA4, LAMAS, LAMB1, LAMC1**, **LAMC2N	LAMB**1, **LAMC2	LAMAS
		MMP14, MMP2
	SEMA3C, SEMA3D, SEMA4A, SEMA4B, SEMA4D, SEMASB, SEMA6C	SEMA4A
ADAMTSL4	ADAMlO, ADAM17	

### Extracellular matrix composition of PDX tumors was altered in response to serial transplantation in murine models

Then we sought to identify genomic features that were required to maintain an intact tumor matrix for in vitro studies. We focused on gene expression changes of matrix-associated genes within TU-BcX-4IC. First, we compared the expression of the human-specific matrix-associated genes that were within the top 100 human genes with increased expression in mouse-transplanted tumors with the matched mouse matrix-associated genes. We observed a pattern in which mouse genes were expressed in complementary patterns to the human genes, with the mouse genes showing increased gene expression when compared to the human genes with reduced expression (Supplementary Fig. S10). This association was more pronounced for some matrix genes (*P4HA2, COL6A2, COL6A1, COL4A2, COL4A1, GPC6, LAMC1, VIM, LGALS3BP, LAMB1, SERPINH1, LGALS3*) and not observed with other genes (*SERPINA3, PCOLCE2, PLAUR, LOXL2, COL6A3, MMP9, COL5A2, BGN*). These data suggest that matrix-associated gene expressions that were lost when human tumors were transplanted in mice, subsequently were expressed by mouse host cells to maintain the ECM structure during serial transplantation.

**Fig. 6:**
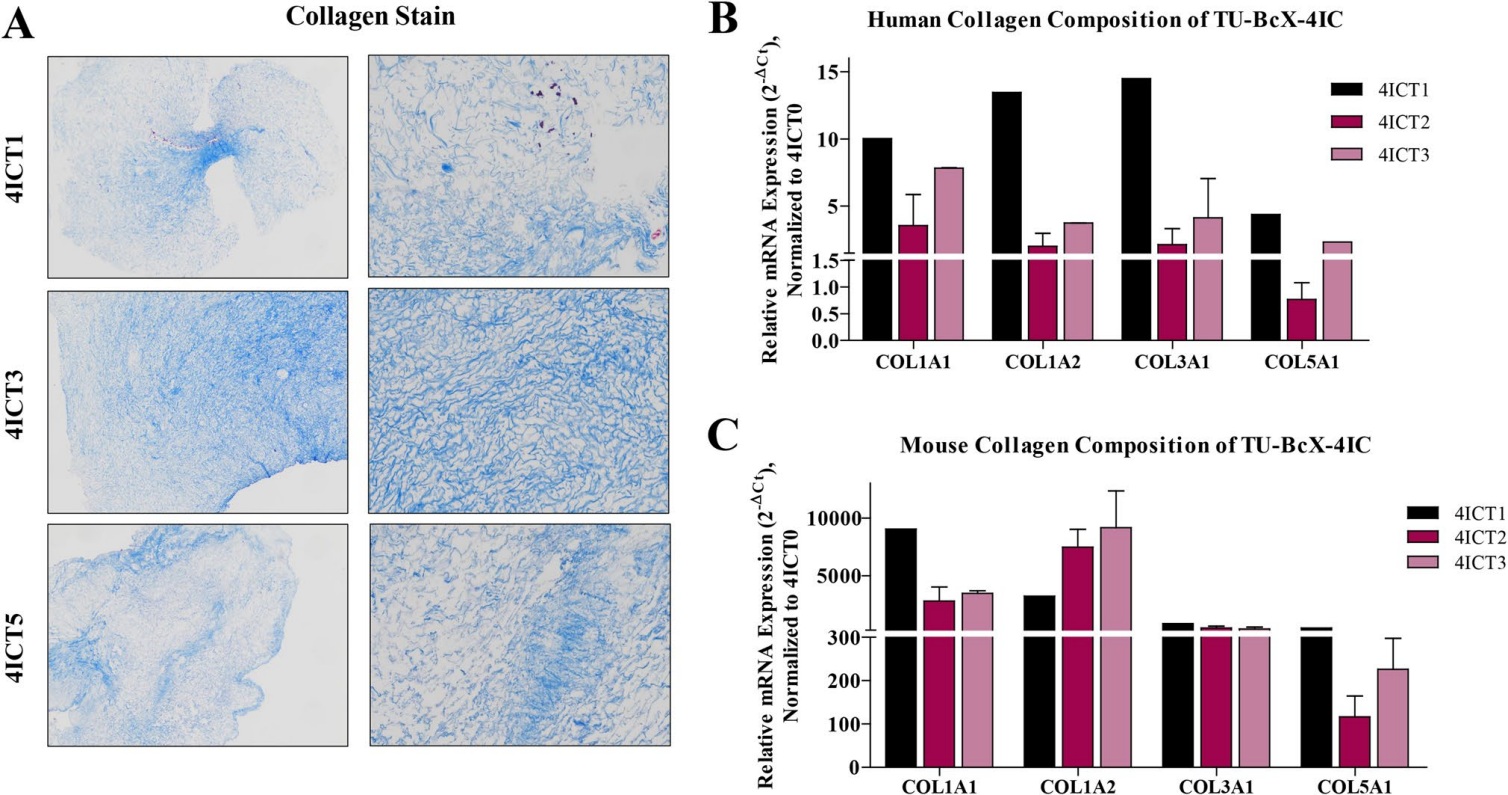
Collagen composition of serially transplanted TU-BcX-4IC PDX tumors. Serially transplanted TU-BcX-4IC PDX tumors were decellularized and consecutive passages of TU-BcX-4IC tumor serially transplanted in mice (T1, T3, T5) are exhibited. **A** Masson’s Trichrome was used to stain decellularized tumors to visualize collagen fiber organization. Decellularized tumors were formalin fixed, paraffin embedded, stained and representative images are shown at 10 × and 100 × magnification. *COL1A1, COL1A2, COL3A1* and *COL5A1* were analyzed using qRT-PCR with both **B** human- and **C** mouse-specific primers. Represented data is normalized to mouse-specific primers to evaluate relative gene expression. Due to the limited availability of tumor tissue, analyses were performed in duplicate, except for T1 and T6 which were single samples only. Error bars represent S.E.M. *p < 0.05, ***p < 0.001

Next, we interrogated the response of PDX serial transplantation to the collagen gene family, based upon our previous observations that collagen network genes were differentially expressed between the original tumor and after serial transplantation in mice. Overall fibrillary collagen composition did not change dramatically, as evident from Masson’s trichrome staining of serially passaged tumors (Fig. 6A). To further demonstrate the change in collagen composition over serial transplants in mice, we compared relative gene expression of human- and mouse-specific collagens using qPCR. Interestingly, we observed a sequential downregulation of human-specific collagens (Fig. 6B). These results were, to some degree, expected,since other studies have shown that one of the greatest obstacles in PDX models is gradual mouse stromal invasion with serial transplantation. Then, we analyzed the collagen gene expression using qPCR with mouse-specific collagen primers. As expected, overall mouse-specific collagen gene expression increased in TU-BcX-4IC tumors that were serially transplanted in mice (T1, T2, T3, T5, T6, T7) compared to the original patient tumor (T0) (Fig. 6C). Consistently, in higher passage PDX tumors, we observed a decrease in human-specific collagen expression when compared to lower passage tumors (COL1A1, COL1A2, COL3A1, COL5A1). We also observed that with higher serial passaged, with respect to COL1A1 and COL1A2 human-specific mRNA expressions, there was a subsequent increase in mouse-specific collagen gene expressions. This was not observed with COL3A1 and COL5A1, suggesting that these collagens derived from mouse cells were not necessary for maintaining tumor growth. Additionally, we did not observe that these collagens were recruited by the mouse stromal cells to compensate for the loss of human-specific COL3A1 and COL5A1. These data suggest that tumor-specific mouse matrix gene expression can be stimulated to compensate for the transcriptional loss of human matrix.

### Serial transplantation of PDX tumors maintain consistent stiffness

**Fig. 7:**
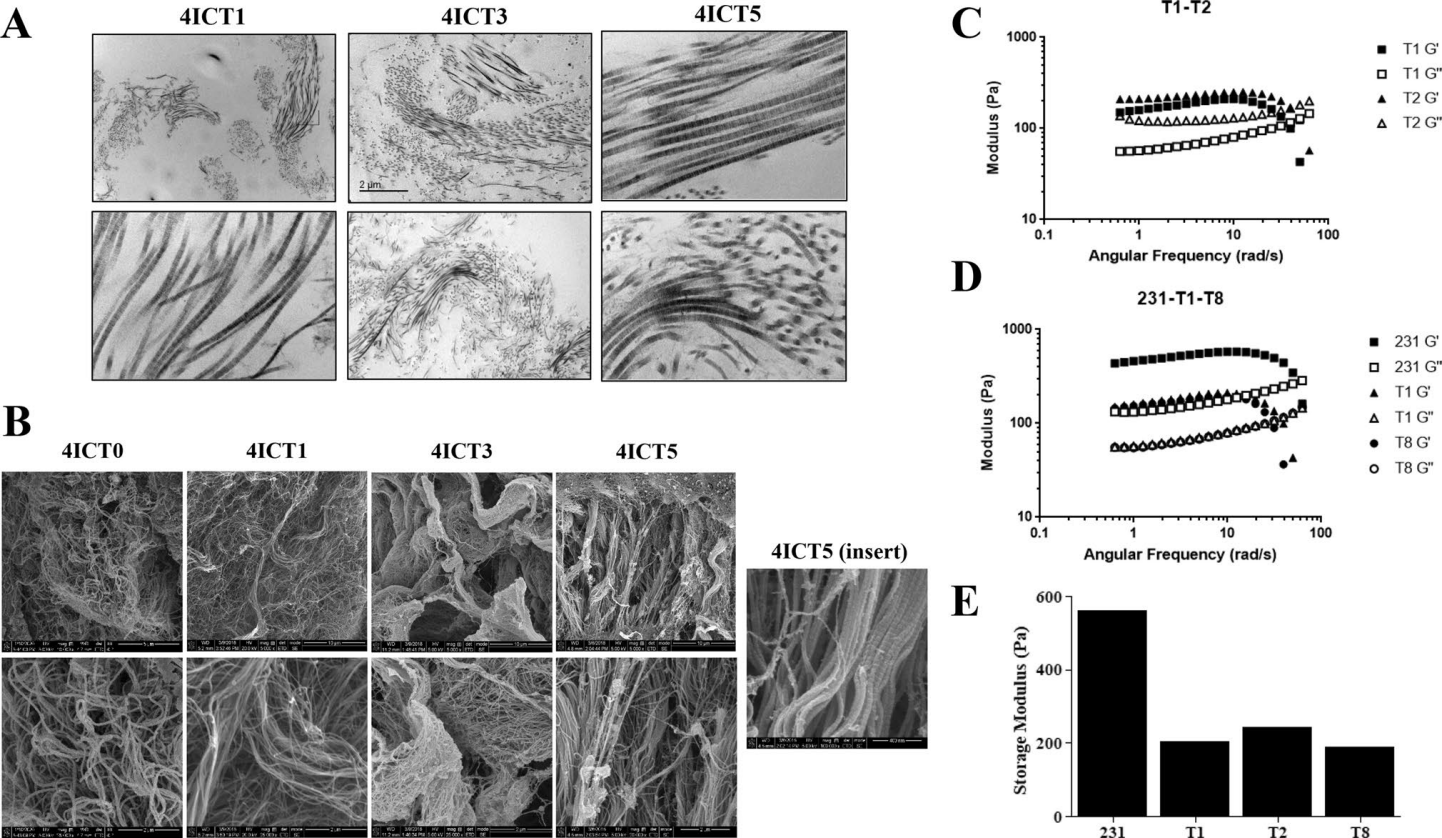
Biophysical properties of TU-BcX-4IC tumors in serial transplantation. **A** Transmitted electron microscopy (TEM) images of decellularized, serially transplanted tumors were visualized to show the organization of collagen fibers. Representative images are shown at 10 × and 100 × magnification. **B** Cryogenic scanning electron microscopy images of serially transplanted TU-BcX-4IC tumors following tissue decellularization. Representative images shown at 5,000X (T1–T5) 10,000X (T0) and 25,000X (T0-T5) magnification. The insert of the TU-BcX-4IC T5 tumor reveals capabilities of the cryo-SEM technique to visualize matrix architecture on a nanometer scale. **C** Rheometry data comparing tumor stiffness of early passage (T1, T2) PDX tumors, and **D** comparing TNBC cell line-derived tumors (MDA-MB-231) to low (T1) and higher (T8) TU-BcX-4IC tumors. Storage modulus (Pa) and angular frequency (rad/s) are displayed. **E** Graph denoting relative storage modulus (a measure of tumor stiffness) across various serial passages and compared to MDA-MB-231 tumors

To determine if this compensation resulted in matrix retention at a structural level, we next sought to visualize and evaluate ECM architecture changes with serial tumor transplantation. This analysis was done independent of cellular composition through the utilization of a novel technique of tissue decellularization [28]. The relative orientation of fiber bundles was observed using transmitting electron microscopy (TEM) (Fig. 7A) and to visualize matrix fibers on a nanoscale level, cryogenic-scanning electron microscopy (cryo-SEM) was employed (Fig. 7B).

Alignment, porosity, and fiber bundles of ECM fibers in decellularized 4IC tumors across serial passages in mice were interrogated. Rheometry testing was performed on decellularized serial tumor transplants to assess if matrix stiffness changes after multiple passages. Results demonstrate that while TU-BcX-4IC primary tumor shows to be stiffer (563 Pa), serial passages T1 (206 Pa), T2 (243 Pa), and T8 (190 Pa) exhibit no significant differences in matrix stiffness (Fig. 7C, D). This correlates with no significant difference in stiffness between earlier and later passages (Fig. 7E). Moreover, comparing results between passages indicate that using a Matrigel™ coating on earlier passages (T1, T2) did not alter stiffness characteristics. Although there were additional passages available to test, the final sample sizes were not large enough and did not meet our requirements for obtaining quality data. Therefore, rheometry data for these passages can be found in Supplementary Figure S11.

## Discussion

Metaplastic breast carcinoma is a rare, clinically aggressive subtype of breast cancer with the capacity to proliferate rapidly and is resistant to current standard treatment options with chemotherapy; patients often present with advanced disease. The diverse heterogeneity within MBC contributes to the difficulty in treating this tumor type with targeted therapies. Constituents, fiber alignment and biophysical properties of the tumor matrix contribute to MBC heterogeneity. Developing translational models to recapitulate and study the tumor matrix accurately enhances understanding of this rare breast cancer subtype [37]. Here we introduce a new PDX model for MBC and provide an extensive in-depth characterization of physical and genomic features of the tumor using a variety of highly innovative and translational techniques. Our work demonstrates that this model can be used to study inherent characteristics of, or drug effects on, a variety of features that contribute to the clinically aggressive phenotype of MBC, including spontaneous metastasis, a high CSC population, and drug resistance.

The rapid tumor take and growth rates exhibited by TU-BcX-4IC tumors are crucial features for a successful PDX model. TU-BcX-4IC spontaneously metastasized to both the lungs and liver of SCID/Beige mice, with detectable circulating mouse and human cfDNA as well as CTC DNA. cfDNA and CTC DNA are prognostic indicators of metastasis in breast cancer, with elevated levels of cfDNA being a more sensitive prognostic indicator of a worse clinical prognosis when compared to the presence of CTCs [30, 31]. Our data further showed that TU-BcX-4IC tumors and cells have prominent CSC populations, although the cells only maintain the CSC population when grown in a system that maintains features of the human breast environment. Here we demonstrate for the first time the translational application of the physiologically relevant BC-MPS system to evaluate CSC populations of human breast tumors in culture conditions.

MBC tumors often do not respond to targeted chemotherapies. Similarly, TU-BcX-4IC cells were resistant to many FDA-approved therapies from the NCI drug set compared to an established TNBC cell line, MDA-MB-231 cells. Specifically, TU-BcX-4IC was most resistant to topoisomerase inhibitors and plant alkaloids, as well as the small molecule inhibitors cabozantinib, cobimetinib and ponatinib. Using an innovative technique for spheroid culture and analysis [25–27] we observed resistance to physiologically relevant doses of paclitaxel (10 nM–100 nM), reflective of the original patient tumor resistance to taxane-based therapy. The Pu·MA system, which uses a vacuum-regulated set-up to change media without disrupting spheres or organoids was employed to visualize and quantify the effects of paclitaxel on TU-BcX-4IC organoids. These data revealed a high EC50 range, further confirming the taxane-resistant phenotype of TU-BcX-4IC. In our drug screening process, we also found drugs that induced a cytotoxic response in TU-BcX-4IC cells, suggesting novel therapies that can be explored as potentially effective in this patient’s tumor.

A limitation of using PDX models is the potential for mouse stromal infiltration after serial transplantation [23]. However, the implications of these observations are not well understood. Recent studies have found that the overall genomic stability of the tumors remain relatively unchanged after serial transplantation, although components of both mouse and human gene expression are altered [37]. Serial passaging of PDX tumors in mice results in mouse stromal infiltration into the tumor, with the percentage of mouse stromal composition not extensively altered with serial passaging [38]. We have confirmed these observations, showing the overall transcriptomic and gene variant analyses from both RNA sequencing were relatively unchanged after serial passaging. However, when we examined specific gene clusters and families associated with the tumor microenvironment, we identified more significant changes in tumor gene expression in those tumors exposed to the murine microenvironment. This demonstrates that even after one serial passage in a murine model, the genomic landscape of tumors is altered.

To evaluate the effect of mouse stromal influence on tumor gene expression, we then aligned the RNA sequencing results separately to both mouse and human genomes. We found that there were more genes uniquely mapped to the mouse genome in higher, serially transplanted tumors. These findings suggest the human tumor cells may have “educated” the mouse cells and stroma so that the mouse gene expression compensated for the genes lost within human tumors upon serial passaging. Similarly, other studies have also demonstrated that some cellular processes, including immune regulation [37] and ECM regulation [39], are lost over serial passaging. Recent studies examining epithelial-mesenchymal transition dynamics and tumor-stromal interactions has questioned the necessity of maintaining human stroma and whether mouse fibroblasts can be “educated” to replace the human stroma [40]. We hypothesized that identifying the specific compensated genes facilitates the identification of genes that are necessary for tumor maintenance, along with those that are deemed expendable.

This pattern was also observed with matrix-associated gene expressions, with reduced human gene expressions in T1 and T4 tumors, and increased mouse-specific expression of the same genes. These data demonstrate preliminary evidence that the transcriptomic composition of PDX tumors essentially changes in response to a foreign microenvironment. The tumors appear to adapt to their new environment by recruiting foreign components to develop the microenvironment required for tumor formation and maintenance. Collagens comprise a large structural component of the extracellular matrix [41]. Based upon our RNA sequencing data, fibrillary-associated collagens (I, III) and collagens associated with TNBC (X) were found to be increased in serial transplantation of tumors. Fat-associated collagens (VI) and basement membrane collagens (IV) were also decreased. One possible reason for these observations is the loss of human adipose over serial transplantation, as were collagens associated with the basement membrane (IV). When we further interrogated the expression of collagen genes commonly expressed in breast cancer ECM (*COL1A1, COL1A2, COL3A1* and *COL5A1*) we observed decreased endogenous gene expression of these collagens in higher passage TU-BcX-4IC tumors compared to lower passage. These data confirm that human ECM gene expression is lost after serial passaging in mice.

These findings were specific to the type of collagen examined. For example, loss of type V human collagen expression was not restored by mouse collagen type V expression, while collagen type I had increased gene expression in both mouse and human. These data suggest type V collagen expression is not necessary for MBC tumor development, while collagen type I appears to be necessary. For example, in physiologic conditions, COL5A1 polymerizes with type I collagen to adjust collagen molecule diameter, with COL5A1 shown to be a potential biomarker for tumor progression in breast cancer [42]. Here we provide preliminary evidence that COL5A1 expression is not necessary for tumor formation in our MBC model, suggesting this collagen may be specific for certain subtypes of breast cancer.

The composition and biophysical properties of ECM within MBC tumors remains under-characterized [22]. We are the first to use tissue decellularization of PDX tumors to visualize and characterize the ECM of human MBC tumors to examine the ECM without the influence of other cellular components. Physical properties of the ECM, including fiber alignment, orientation and even ECM pore size, affect tumorigenesis and metastasis of solid tumor types [43, 44]. Stromal remodeling occurs with advanced disease and in regions where collagen bundles align perpendicular to the tumor boundary. Additionally, tumor cells preferentially invade along the straightened collagen fibers to promote intravasation [45–47]. Cryo-SEM and TEM can be utilized for an in-depth examination of the three-dimensional properties of the ECM, on a nanometer scale.

Although other studies utilizing genomic sequencing to show that human ECM is lost over serial passaging in mice, we are the first to use cryo-SEM and TEM microscopy to visualize this evolution. First, TEM was employed to show that collagen bundle density remained similar throughout serial passaging in mice. In addition to matrix composition and topographical features, the rheometry results confirmed relatively unchanged matrix stiffness in PDX tumors after multiple passages, and similarity in stiffness between earlier and later passages. This suggests that the physical characteristics between serial transplants remain consistent, despite the mouse stromal infiltration into the tumor over time. Therefore, these results further support the notion that using PDX tumor from later passages can accurately investigate how physical and topographical features can impact cell–matrix interactions and therapeutic treatment. Importantly, our rheometry findings also demonstrate that intact decellularized TU-BcX-4IC tumors were stiffer than MDA-MB-231 cell-derived xenografts, supporting the hypothesis that using TU-BcX-4IC tumor scaffolds in in vivo studies more accurately represents important biophysical properties of human tumors compared to cell-derived xenografts. To the best of our knowledge, we are the first group to evaluate serial passaged PDX through tissue decellularization and advanced microscopy to generate 3D tumor models of MBC.

## Conclusions

Models representing rare cancer subtypes are limited by tissue availability. In this study we provided an in-depth characterization of a new PDX model for MBC using innovative and translational techniques and demonstrated viable biologic targets can be discovered using these methods. We demonstrated that human tumors respond and adapt to their new microenvironments by educating the foreign stroma for proper tumor formation, growth, and maintenance. Our data suggest serial transplantation of PDX in murine models may cause genetic drift and differences in tumor populations but enrich for tumor matrix. The decellularized scaffold of higher passage PDX models provides a novel platform to study cell–matrix and cell-stromal interactions within a physiologically relevant system.

## Supplementary Information

Below is the link to the electronic supplementary material.

Supplementary file1 (DOCX 709 kb)

Supplementary file2 (DOCX 46 kb)

Supplementary file3 (DOCX 54 kb)

Supplementary file4 (DOCX 23 kb)

Supplementary file5 (DOCX 386 kb)

Supplementary file6 (DOCX 171 kb)

Supplementary file7 (DOCX 390 kb)

Supplementary file8 (DOCX 442 kb)

Supplementary file9 (DOCX 37 kb)

Supplementary file10 (DOCX 83 kb)

Supplementary file11 (DOCX 211 kb)

## Data Availability

The datasets analyzed during the current study are available from the corresponding author on reasonable request.
