# Supplementary material for: In-depth characterization of a new patient-derived xenograft model for metaplastic breast carcinoma to identify viable biologic targets and patterns of matrix evolution within rare tumor types
Source: Clin Transl Oncol. 2021 Aug 9;24(1):127–44. doi: 10.1007/s12094-021-02677-8 (PMC8732292; doi:10.1007/s12094-021-02677-8)
Supplement: Supplementary file 1 — Supplementary file1 (DOCX 709 kb) [file 12094_2021_2677_MOESM1_ESM.docx]

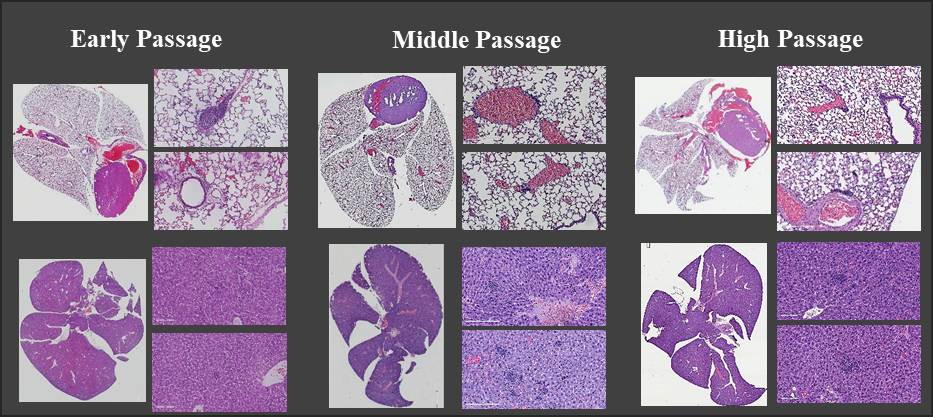


**Supplementary Figure S1.** Lungs and livers harvested and H&E stained from TU-BcX-4IC tumors serially transplanted in murine models. TU-BcX-4IC tumors were implanted in the mfp of SCID/Beige mice. Organs were harvested when tumors reached a maximal volume and were serially transplanted.
