# Supplementary material for: In-depth characterization of a new patient-derived xenograft model for metaplastic breast carcinoma to identify viable biologic targets and patterns of matrix evolution within rare tumor types
Source: Clin Transl Oncol. 2021 Aug 9;24(1):127–44. doi: 10.1007/s12094-021-02677-8 (PMC8732292; doi:10.1007/s12094-021-02677-8)
Supplement: Supplementary file 2 — Supplementary file2 (DOCX 46 kb) [file 12094_2021_2677_MOESM2_ESM.docx]

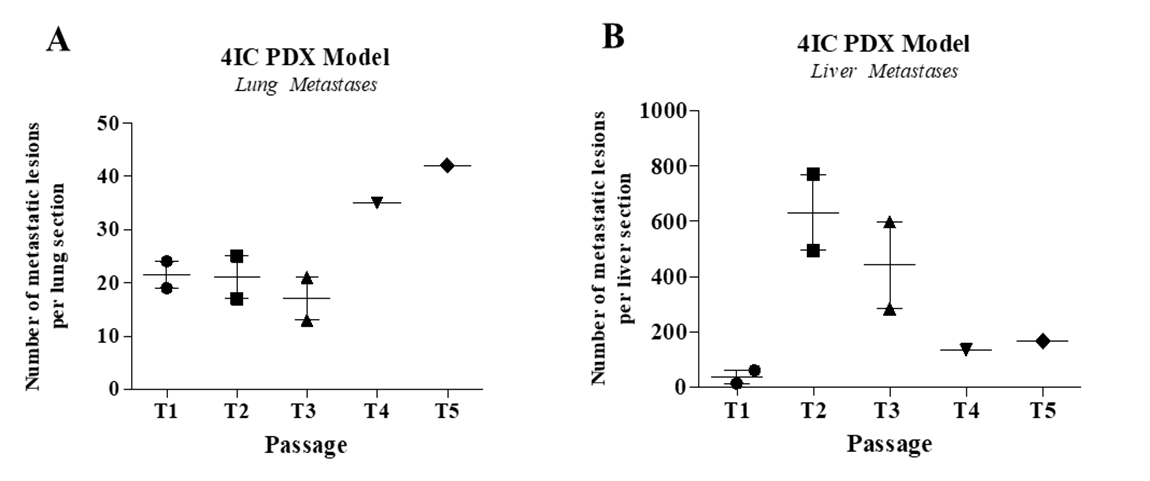


**Supplementary Figure S2.** Lungs and livers were harvested after serial passaging of TU-BCx-4IC in SCID/Beige mice. Organs were fixed, paraffin-embedded, sectioned and stained with H & E to observe metastases. Quantification of lung metastases showed that (A) number of metastases per lung section were consistent over serial transplantation. Quantification of liver metastases showed that (B) number of metastases per liver section were consistent over serial transplantation.
