# Supplementary material for: In-depth characterization of a new patient-derived xenograft model for metaplastic breast carcinoma to identify viable biologic targets and patterns of matrix evolution within rare tumor types
Source: Clin Transl Oncol. 2021 Aug 9;24(1):127–44. doi: 10.1007/s12094-021-02677-8 (PMC8732292; doi:10.1007/s12094-021-02677-8)
Supplement: Supplementary file 3 — Supplementary file3 (DOCX 54 kb) [file 12094_2021_2677_MOESM3_ESM.docx]

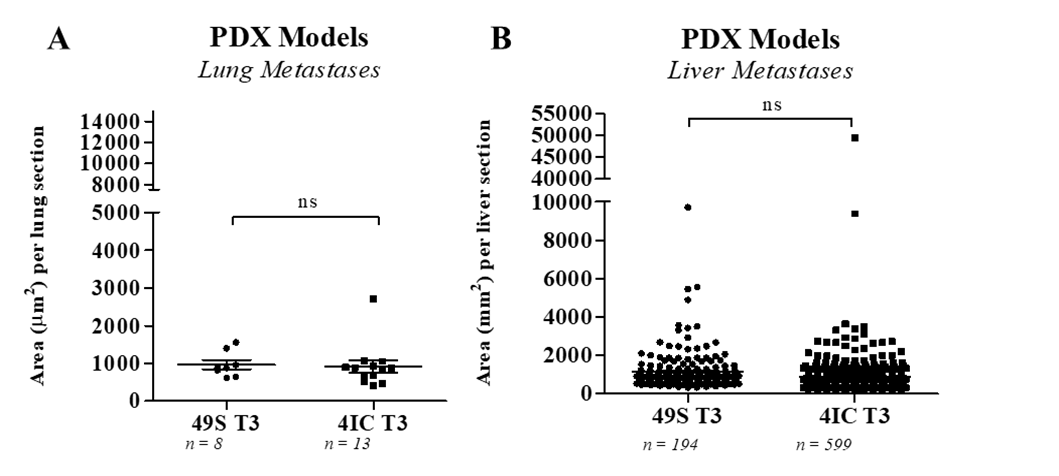


**Supplementary Figure S3.** Comparison of spontaneous metastases of TU-BcX-4IC and TU-BcX-49S tumors that were serially transplanted three times (T3) in SCID/Beige mice. Both TNBC PDX models exhibited consistent metastases to the (A) lungs and (B) livers of mice. One mouse per group was analyzed. Lungs and livers were harvested after serial passaging of TU-BCx-4IC in SCID/Beige mice. Organs were fixed, paraffin-embedded, sectioned and stained with H & E to observe metastases.
