# Supplementary material for: In-depth characterization of a new patient-derived xenograft model for metaplastic breast carcinoma to identify viable biologic targets and patterns of matrix evolution within rare tumor types
Source: Clin Transl Oncol. 2021 Aug 9;24(1):127–44. doi: 10.1007/s12094-021-02677-8 (PMC8732292; doi:10.1007/s12094-021-02677-8)
Supplement: Supplementary file 4 — Supplementary file4 (DOCX 23 kb) [file 12094_2021_2677_MOESM4_ESM.docx]

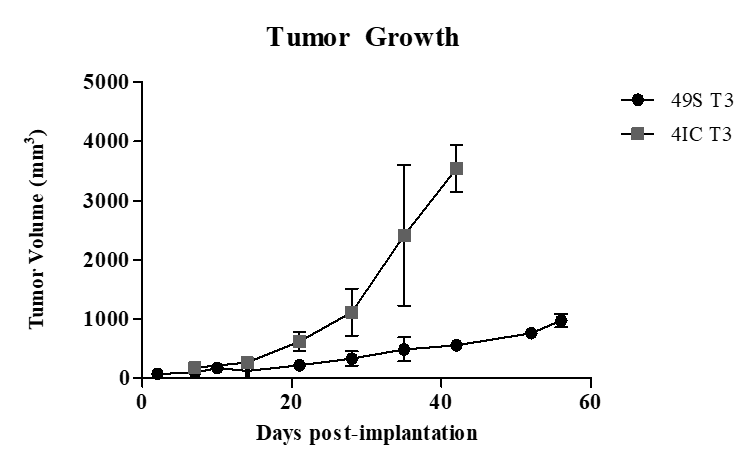


**Supplementary Figure S4.** Comparison of tumor growth of the two TNBC PDX models used in the experiment evaluating CTC cells in the peripheral blood of mice. Black data points represent TU-BcX-49S and grey data points represent TU-BcX-4IC. At the time of peripheral blood harvest, both PDX tumors were serially transplanted in mice three times (T3).
