# Supplementary material for: In-depth characterization of a new patient-derived xenograft model for metaplastic breast carcinoma to identify viable biologic targets and patterns of matrix evolution within rare tumor types
Source: Clin Transl Oncol. 2021 Aug 9;24(1):127–44. doi: 10.1007/s12094-021-02677-8 (PMC8732292; doi:10.1007/s12094-021-02677-8)
Supplement: Supplementary file 5 — Supplementary file5 (DOCX 386 kb) [file 12094_2021_2677_MOESM5_ESM.docx]

**
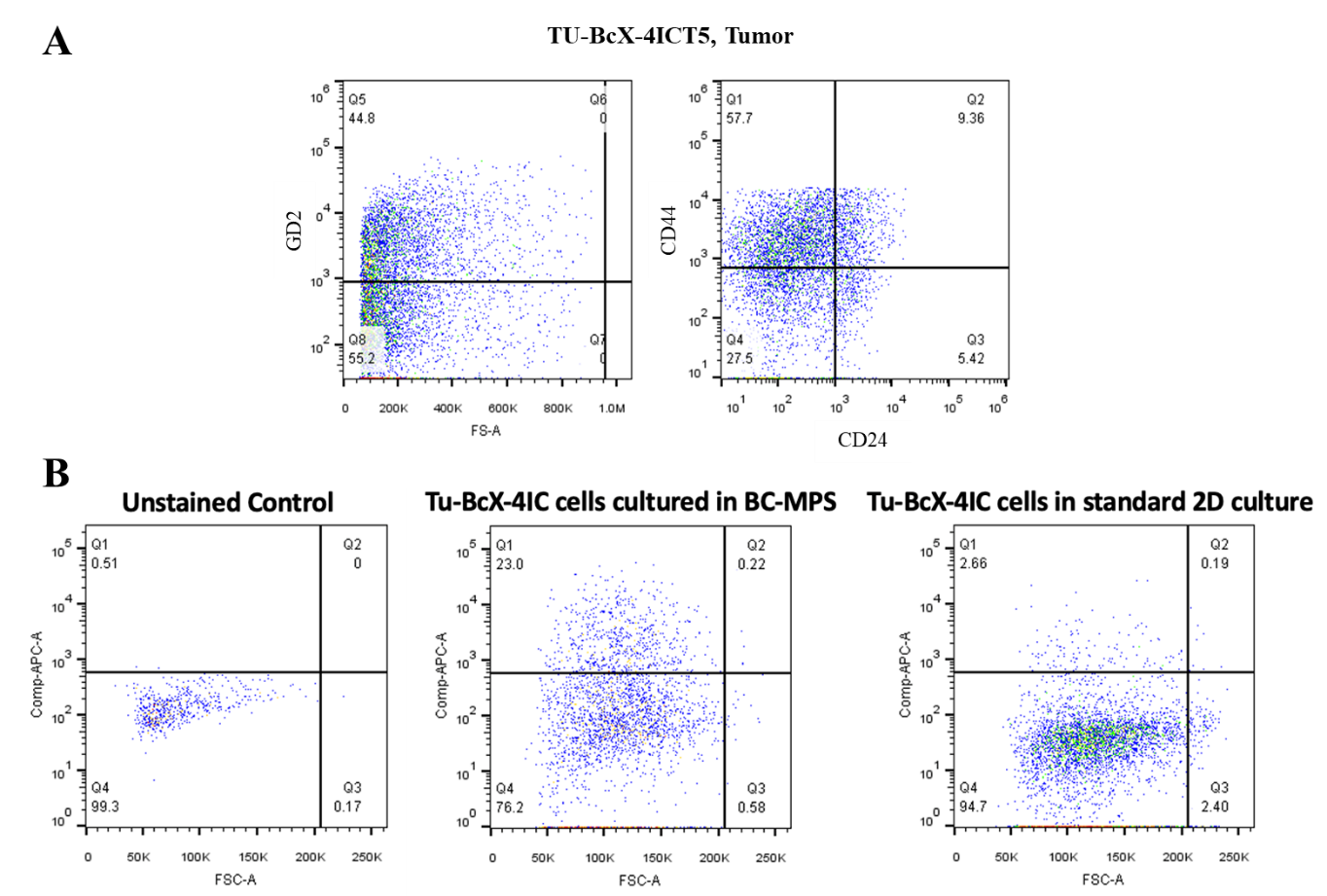
**

**Supplementary Figure S5. Characterization of cancer stem cell-like phenotype within TU-BcX-4IC tumors and cells.** CSC populations are represented as GD2^+^ or CD24^low^CD44^high^. (A) Flow cytometry for CSC populations in TU-BcX-4IC tumors after one serial transplantation in a murine model. (B) Dot plots from the unstained control, TU-BcX-4IC cells cultured in BC-MPS, and cells cultured in the standard adherent conditions stained with GD2 (Comp-APC-A) vs. Forward Scatter (FSC-A). BC-MPS cultured TU-BcX-4IC cells show 23% positive stain for CSCs, while cells cultured in adherent conditions had 2.66% positive stain for CSCs.
