# Supplementary material for: In-depth characterization of a new patient-derived xenograft model for metaplastic breast carcinoma to identify viable biologic targets and patterns of matrix evolution within rare tumor types
Source: Clin Transl Oncol. 2021 Aug 9;24(1):127–44. doi: 10.1007/s12094-021-02677-8 (PMC8732292; doi:10.1007/s12094-021-02677-8)
Supplement: Supplementary file 6 — Supplementary file6 (DOCX 171 kb) [file 12094_2021_2677_MOESM6_ESM.docx]

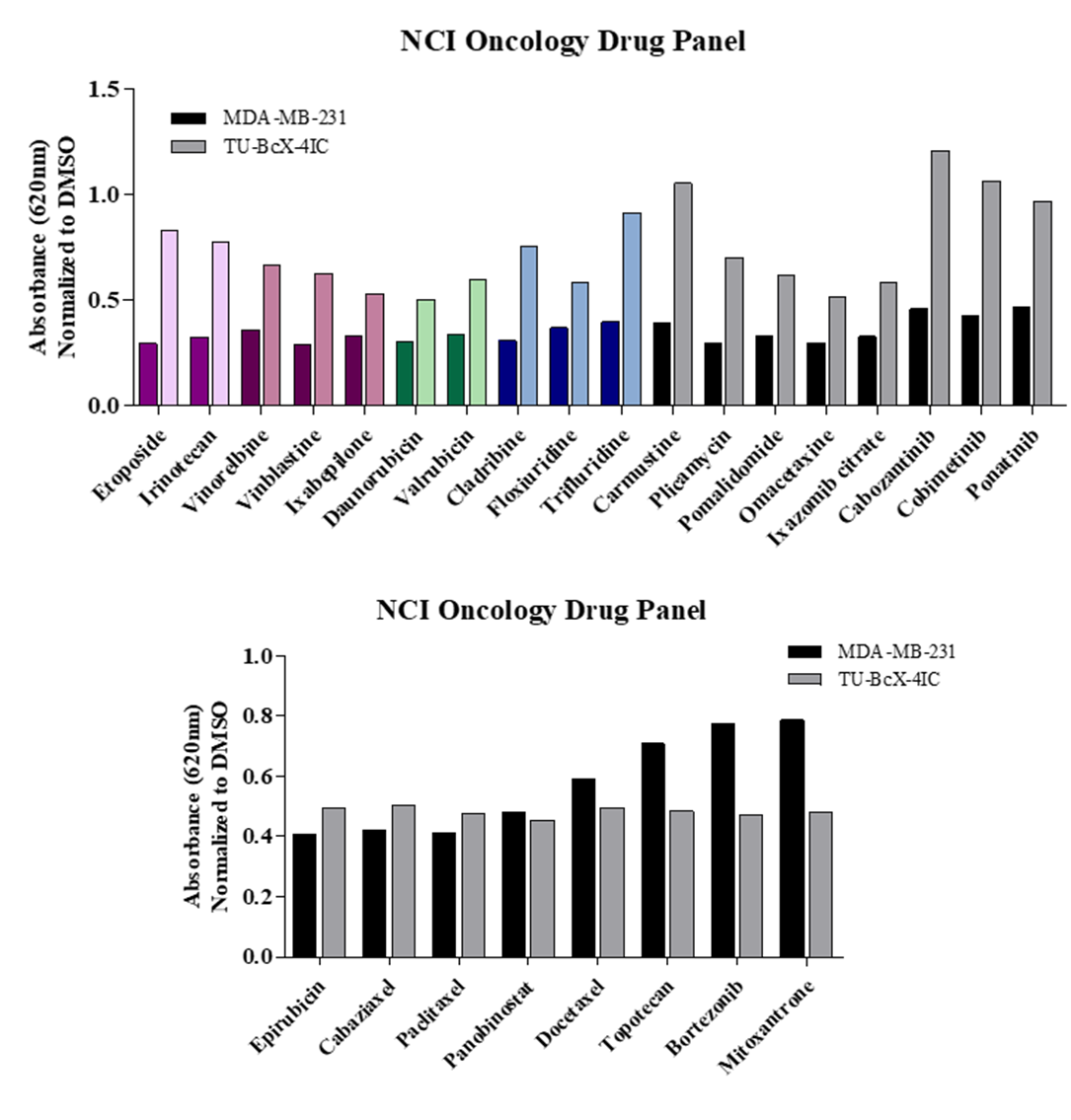


**Supplementary Figure S6.** Quantification of data obtained from the NCI-approved oncology drug screen in MDA-MB-231 and TU-BcX-4IC cells. Crystal violet-stained plates were lysed and absorbance was measured using a spectrophotometer to evaluate relative abundance. Colors in the graphs resemble groupings of drug classes. Only drugs that were either resistant to MDA-MB-231 cells and/or TU-BcX-4IC cells were selected for quantification studies. Black bars represent MDA-MB-231 cells and grey bars represent TU-BcX-4IC cells.
