# Supplementary material for: In-depth characterization of a new patient-derived xenograft model for metaplastic breast carcinoma to identify viable biologic targets and patterns of matrix evolution within rare tumor types
Source: Clin Transl Oncol. 2021 Aug 9;24(1):127–44. doi: 10.1007/s12094-021-02677-8 (PMC8732292; doi:10.1007/s12094-021-02677-8)
Supplement: Supplementary file 8 — Supplementary file8 (DOCX 442 kb) [file 12094_2021_2677_MOESM8_ESM.docx]

**
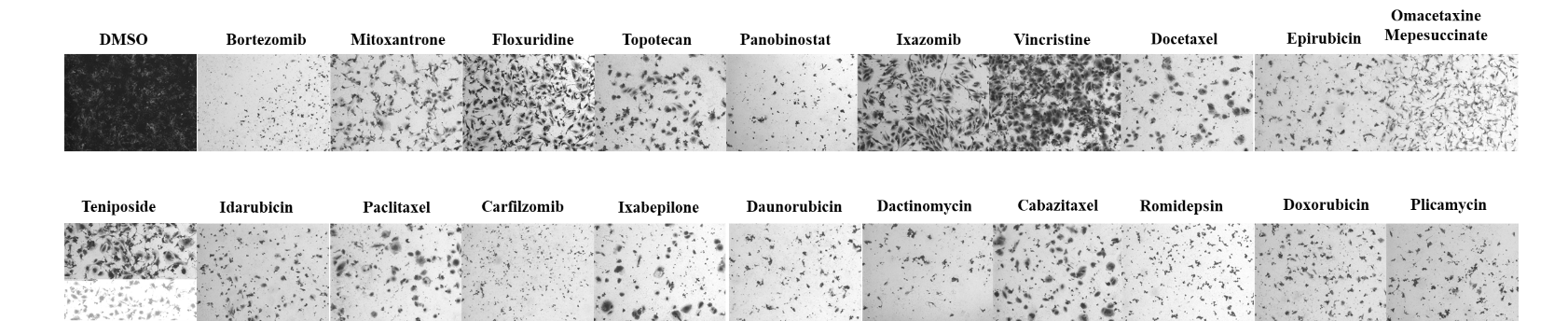
**

**Supplementary Figure S8.** Drugs from the NCI-approved oncology drug set that were cytotoxic to TU-BcX-4IC cells, compared to the DMSO vehicle control. All drugs were screened at 1 µM concentrations.
