# Supplementary material for: In-depth characterization of a new patient-derived xenograft model for metaplastic breast carcinoma to identify viable biologic targets and patterns of matrix evolution within rare tumor types
Source: Clin Transl Oncol. 2021 Aug 9;24(1):127–44. doi: 10.1007/s12094-021-02677-8 (PMC8732292; doi:10.1007/s12094-021-02677-8)
Supplement: Supplementary file 9 — Supplementary file9 (DOCX 37 kb) [file 12094_2021_2677_MOESM9_ESM.docx]

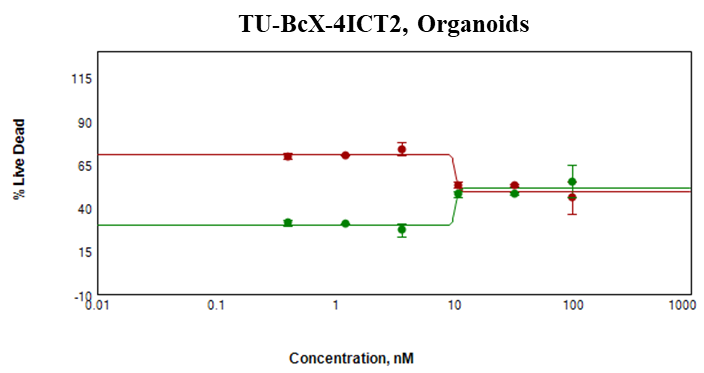


**Supplementary Figure S9.** Quantification of live/dead cytotoxicity stain of TU-BcX-4IC organoids treated with paclitaxel. EC50 range was determined from this quantification, with a range of 6.8-29.4 µM. Red = dead cells, stained with Ethidium III; Green = live cells, stained with Calcein AM.
