# Supplementary material for: In-depth characterization of a new patient-derived xenograft model for metaplastic breast carcinoma to identify viable biologic targets and patterns of matrix evolution within rare tumor types
Source: Clin Transl Oncol. 2021 Aug 9;24(1):127–44. doi: 10.1007/s12094-021-02677-8 (PMC8732292; doi:10.1007/s12094-021-02677-8)
Supplement: Supplementary file 10 — Supplementary file10 (DOCX 83 kb) [file 12094_2021_2677_MOESM10_ESM.docx]

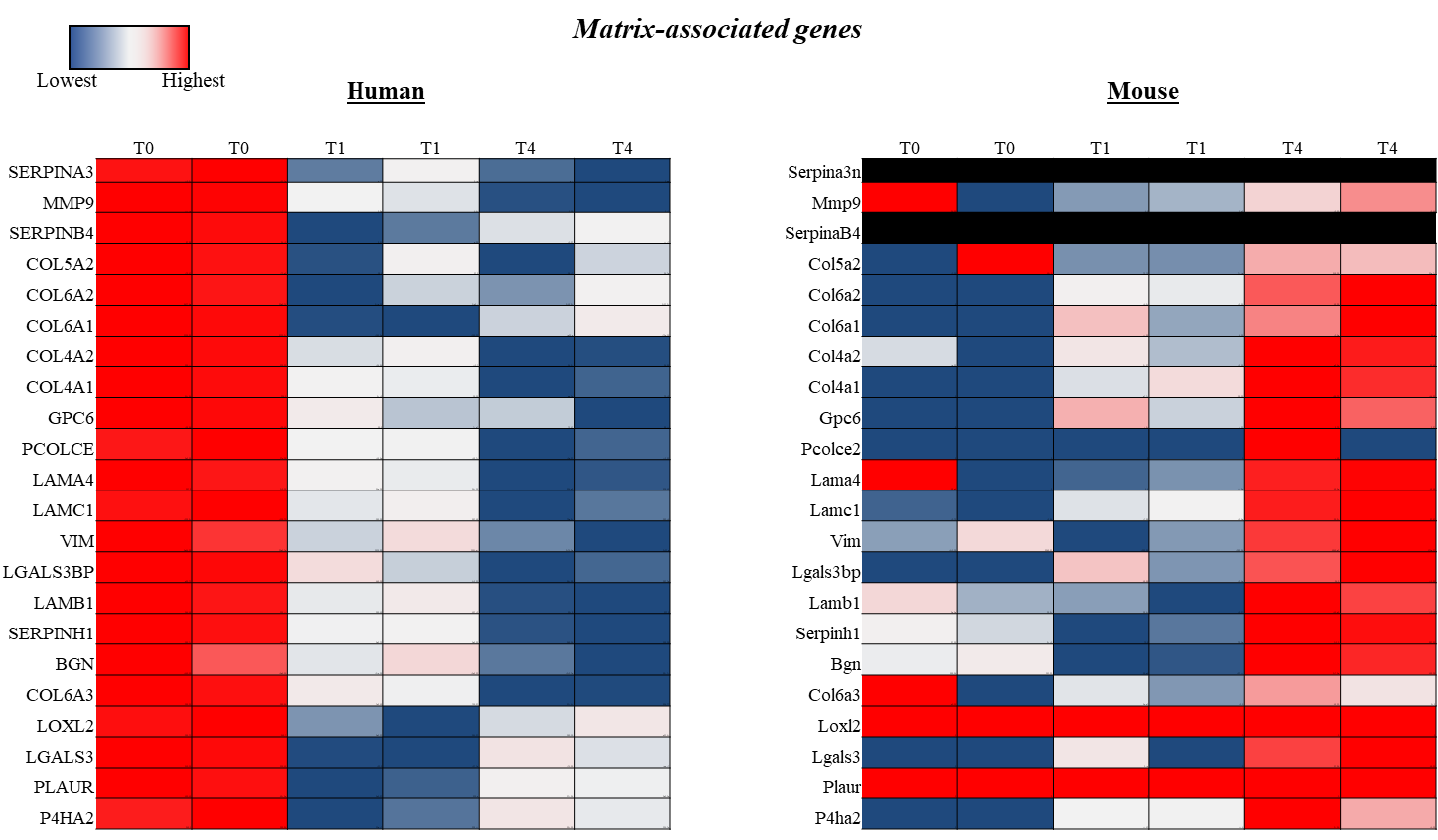


**Supplementary Figure S10.** Matrix associated genes identified from RNA sequencing that were aligned to human or mouse genomes. Data is represented by a heat map for each gene listed, with blue as the lowest value within a gene group and red as the highest value. Each heat map scale for specific genes (T0, T1, T4 passages) are analyzed separately to represent changes in gene expression over serial transplantation; for example, the heat map range for SERPINA3 is unique from the other genes.
