# Supplementary material for: In-depth characterization of a new patient-derived xenograft model for metaplastic breast carcinoma to identify viable biologic targets and patterns of matrix evolution within rare tumor types
Source: Clin Transl Oncol. 2021 Aug 9;24(1):127–44. doi: 10.1007/s12094-021-02677-8 (PMC8732292; doi:10.1007/s12094-021-02677-8)
Supplement: Supplementary file 11 — Supplementary file11 (DOCX 211 kb) [file 12094_2021_2677_MOESM11_ESM.docx]

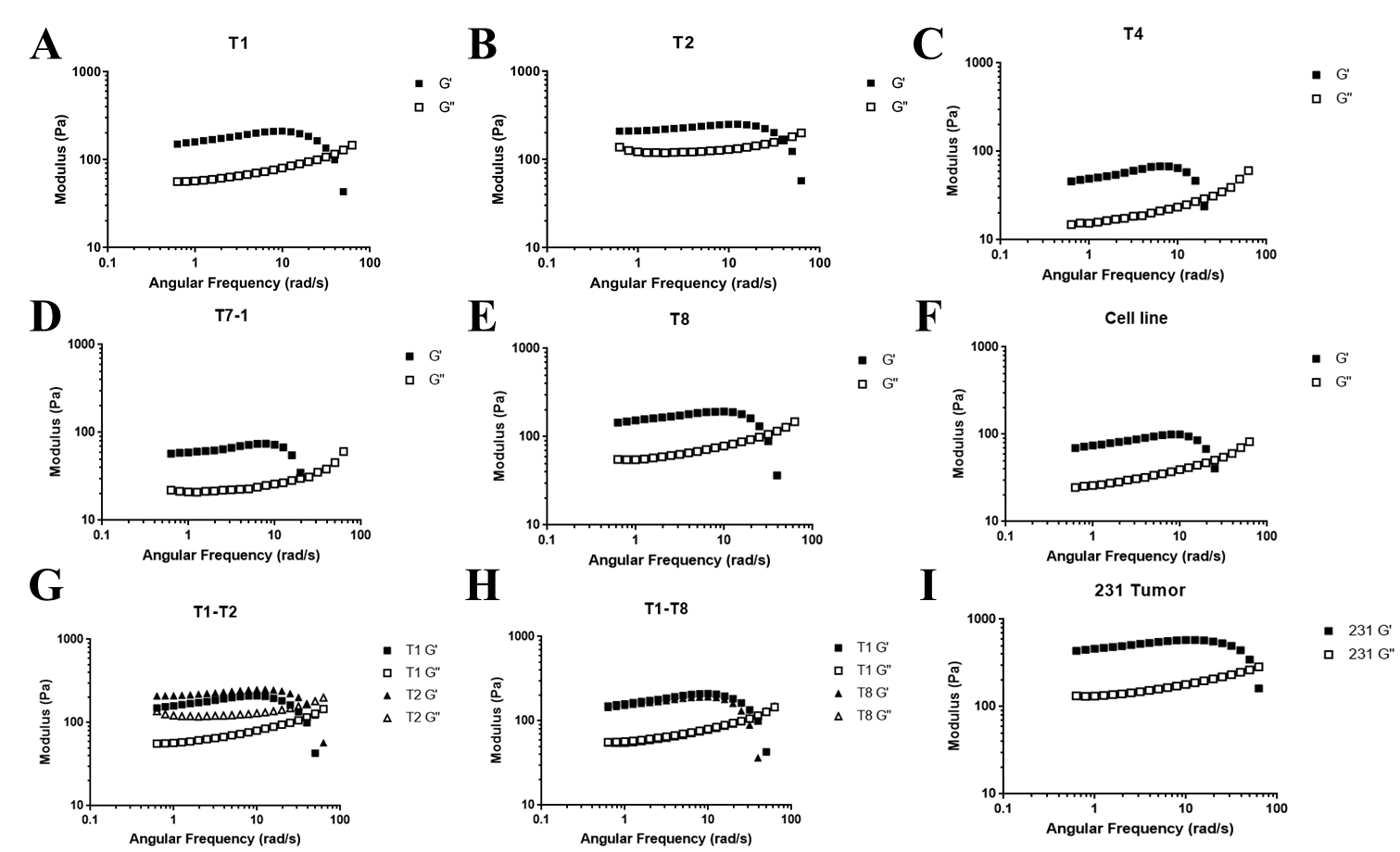


**Supplementary Figure S11. Tumor stiffness of TU-BcX-4IC tumors is consistent through serial passages.** Rheometry data comparing tumor stiffness measured by storage modulus (Pa) and angular frequency (rad/s) of (A, B) early passage (T1, T2), (C) moderately high passage (T4) and (D, E) higher passage (T7, T8) TU-BcX-4IC tumors. (F) Tumor stiffness of tumor derived from TU-BcX-4IC cells implanted in a mouse with Matrigel^TM^. Overlay of (G) lower passage PDX tumors and (H) comparison of lower passage to high passage PDX tumors. (I) Rheometry graph of tumor derived from MDA-MB-231 cells injected into a mouse.
